# Supplementary material for: Effects of health literacy, screening, and participant choice on action plans for reducing unhealthy snacking in Australia: A randomised controlled trial
Source: PLoS Med. 2020 Nov 3;17(11):e1003409. doi: 10.1371/journal.pmed.1003409 (PMC7608866; doi:10.1371/journal.pmed.1003409)
Supplement: S1 Surveys — (PDF) [file pmed.1003409.s002.pdf]

S2 Surveys: Baseline and follow-up surveys

Note: Some images have been removed to preserve copyright.
Headings shown in maroon were not visible to participants; these are included to help navigate the survey document.

Baseline survey

Smart snacking

We are doing a research study to find out more about tools to help people eat less unhealthy snacks. While many of us want to change the way we snack, this can be very hard. Often we make plans but have trouble sticking to them over long periods of time. This study will look at online tools that help people stick to their plans.

What will I be asked to do?

If you decide that you want to be in our study, we will ask you to:

1. Complete questions online (for example, about foods you eat)
2. Use the online planning tool to create a plan to help you eat less unhealthy snacks. We will ask you to follow the plan for 4 weeks.
3. You will receive 3 reminder messages about your plan during that time.
4. Complete questions online after 4 weeks.

To read more information about this study, please click here <link to PDF version of PIS>.

Demographic section

Before we start, please answer these questions

Age in years _______________

Gender

Male	Female	Other

What is your height in **cm**? _____________

What is your weight in **kg**? _____________

Do you have diabetes

No	Yes, Type 2 diabetes	Yes, Gestational diabetes	Yes, Type 1 diabetes

Do you speak English at home?

Yes	No

[If 'No" is selected:]

What language(s) do you speak at home?

Arabic	Cantonese	Mandarin	Hindi	Filipino/Tagalog
Korean	Tamil	Punjabi	Spanish	Italian

Turkish	Other (please write the language in the box)			

What is your highest level of education?

Less than high school	Certificate III or IV
High school graduate	Diploma, Bachelor or equivalent
Certificate I or II	Masters or Doctoral degree, or equivalent

What is your employment status?

Employed full time	Retired
Employed part time	Student
Unemployed looking for work	Disabled
Unemployed not looking for work	

[If they answered that they have type 2 diabetes]
Do you use insulin (Yes/no)

What year were you diagnosed with diabetes? _______________

Smart snacking introduction

Smart snacking

Snacks are important because they keep us going until the next meal. Sometimes though, we eat too many, or choose snacks that are unhealthy.

Even though each snack is usually small, over time the snacks add up. This can make us gain weight.

Smart snacking means choosing nutritious, healthy snacks that give you energy until the next meal.

Which snacks are healthy?

Healthy snacks are low in kilojoules, fat, salt and sugars. These include fresh fruit, vegetables with dup, small amounts of dried fruit or nuts, yoghurt, coffee made with low fat milk, raisin toast, rice cracks and corn things.

Which snacks are unhealthy?

Unhealthy snacks are high in kilojoules, fat, salt and sugars. These include biscuits, cheese crackers, cakes, muffins, pastries, chocolate, lollies, potato chips, hot chips, French fries, some muesli bars and large coffees made with full cream milk.

Before we get started on smart snacking, we'd like to know a little more about your snacking habits.

Place your mouse over any words that have a dotted line to see what they mean! Try it out on the words above.

[Mouse-over words shown in brackets below]

Habit strength for unhealthy snacking

Think about your snacking habits in the **last month**. Choose a button for each of the questions below.

	Strongly disagree	Disagree	Disagree a bit	Neither agree nor disagree	Agree a bit	Agree	Strongly agree
1. I eat unhealthy snacks frequently (all the time)	☐	☐	☐	☐	☐	☐	☐
2. I eat unhealthy snacks automatically (without thinking)	☐	☐	☐	☐	☐	☐	☐
3. I eat unhealthy snacks without having to consciously remember (When I eat unhealthy snacks I have not made an active decision) to eat them.	☐	☐	☐	☐	☐	☐	☐
4. I feel weird if I do not eat unhealthy snacks	☐	☐	☐	☐	☐	☐	☐
5. I eat unhealthy snacks without thinking	☐	☐	☐	☐	☐	☐	☐
6. It would require effort not to eat unhealthy snacks	☐	☐	☐	☐	☐	☐	☐
7. Unhealthy snacks belong to are part of my routine (what I usually do)	☐	☐	☐	☐	☐	☐	☐
8. I start eating unhealthy snacks before I realise I'm doing it	☐	☐	☐	☐	☐	☐	☐
9. I would find it hard not to eat unhealthy snacks	☐	☐	☐	☐	☐	☐	☐
10. I don't need to think about eating unhealthy snacks)	☐	☐	☐	☐	☐	☐	☐
11. Unheathy snacking is typical normal for me	☐	☐	☐	☐	☐	☐	☐
12. I've been eating unhealthy snacks for a long time	☐	☐	☐	☐	☐	☐	☐

Extent of perceived unhealthy snacking

Think about your snacking habits in the **last week**. To what extent (How true is it that your snacks this week were) have your snacks this week been:

	Not at all				Very much			
	0	1	2	3	4	5	6	7
**healthy** (for example: apple, banana, or dried fruit)								
**unhealthy** (for example: chocolate, crisps or cake)								

Snacking scores (main outcome)

How often do you usually eat oven baked potato gems/chips/hashbrowns, hot chips / French fries, wedges or fried potatoes?

Each day	Each week	Each month	I don't eat this

In totally, how many serves of potato gems/chips/hashbrowns, hot chips / French fries, wedges or fried potatoes do you usually eat in the timeframe selected above?

1 serve =

- 12 fried hot chips
- 1 cup (60g) potato gems / hashbrowns, or wedges

How many servings? (0 to 10)

The same questions will also be asked for the following foods:

- savoury snacks such as crisps, pretzels or plain/flavoured crackers?
    - 1 serve = 1/2 snack size packet of crisps 1 handful (30g) of salty crackers or pretzels
- sweet biscuits/cakes/ buns/ muffins/ doughnuts? Include both home-made and bought.
    - 1 serve = 2-3 (35g) sweet biscuits / 1 donut / 1 slice (40g) of plain cake or sweet bun / 1 small muffin
- savoury pastries? This includes pies, pasties, sausage rolls, Kransky Dogs and frankfurters wrapped in pastry.
    - 1 serve = 1/4 (60g) commerical meat pies or pastie / 1 party size pie or sausage roll
- snack type bars? This includes muesli bars, fruit bars and breakfast cereal bars. *(1 bar = 1 serve)*
- chocolate or lollies? Include all types of chocolate and both hard and soft lollies.
    - 1 serve = ½ chocolate bar /4 pieces of chocolate (25g) /5-6 (40g) lollies
- ice-cream or ice-blocks? This includes ice-blocks, ice-cream in a bowl or ice-creams on a stick.
    - 1 serve = 2 scoops (60g) ice-cream / 1 stick ice-cream or ice-block
- soft drink, cordial or sports drinks? Include all drinks with **added sugar** such as soft drinks, cordials, fruit drinks, vitamin waters, energy and sports drinks.
    - 1 serve = 1 can (375mL of soft drink) / 1 bottle of sports drink / 375mL of cordial or fruit drink
- How often do you usually eat meat products? Include sausages, frankfurters, devon, fritz, ham, salami, hot dogs, hamburgers and chicken nuggets
    - 1 serve = 2 slices (55g) processed meat such as ham, salami, devon or fritz / 2 thin of 1 ½ thick (60g) sausages, frankfurters or hot dogs / 1 hamburger pattie / 3 (60g) chicken nuggets

Intentions to reduce unhealthy snacking (baseline)

What do you think about snacking?

Over the next month...

	Strongly disagree	Disagree	Disagree a bit	Neither agree nor disagree	Agree a bit	Agree	Strongly agree

I want to eat fewer (less) unhealthy snacks	☐	☐	☐	☐	☐	☐	☐
I plan to eat fewer unhealthy snacks	☐	☐	☐	☐	☐	☐	☐
I intend (plan) to eat fewer unhealthy snacks	☐	☐	☐	☐	☐	☐	☐

Need for cognition

For each of the following statements please indicate to what extent they apply to you. Please note that there are no right or wrong answers

	Strongly disagree	Disagree	Disagree a bit	Neither agree nor disagree	Agree a bit	Agree	Strongly agree
I like to have the responsibility of handling a situation that requires a lot of thinking	☐	☐	☐	☐	☐	☐	☐
I would rather do something that requires little thought than something that is sure to challenge my thinking abilities	☐	☐	☐	☐	☐	☐	☐
I try to anticipate and avoid situations where there is likely to be a chance that I have to think deeply about something	☐	☐	☐	☐	☐	☐	☐

Newest Vital Sign (health literacy)

[Newest Vital Sign nutrition label image, see https://www.pfizer.com/health/literacy/public-policy-researchers/nvs-toolkit]

1. If you eat the entire container, how many calories will you eat? (Do not use any commas, spaces or decimals) _____________
2. If you are allowed to eat 60 grams of carbohydrates as a snack, how much ice cream could you have?

Amount (Use a number. Half can be written as '0.5')	Unit of measurement
____________________	Drop down box with options: cup(s)/container(s)/serve(s)

3. Your doctor advises you to reduce the amount of saturated fat in your diet. You usually have 42 grams of saturated fat each day, which includes one serving of ice cream. If you stop eating ice cream, how many grams of saturated fat would you be consuming each day?
Grams: ___________________________
4. If you usually eat 2,500 calories in a day, what percentage of your daily value of calories will you be eating if you eat one serving?
Per cent: _________________

Pretend that you are allergic to the following substances: penicillin, peanuts, latex gloves, and bee stings.
5. Is it safe for you to eat this ice cream? (Yes/No)
6. Why not? ______________________

Health literacy single-item screener

How confident are you filling out medical forms by yourself?

- ☐ Not at all
- ☐ A little bit
- ☐ Somewhat
- ☐ Quite a bit
- ☐ Extremely

Allocation method: All participants

Great! It looks like you're ready to **snack smarter**! The next step is to come up with a plan.

Allocation method: Random and preference assessed (Arm A1)

We have two tools that can help you come up with a plan. Click on the tool you would like to use.

You may not be given this tool to use, but we would like to know which one you prefer.
[in this and the other example below, 101 and Pro order was randomised]

- **Smart snacking 101:** helps you create a simple plan using common ways to eat less snacks
- **Smart snacking Pro:** helps you create a tailored plan that suits your specific situation
- **I am not sure which tool I prefer**

[if participant clicks 'I am not sure which tool I want']:
Here is some more information about the tools.

- **Smart snacking 101:** gives ideas about ways that people often eat less snacks, so that you can come up with your own simple plan
- **Smart snacking Pro:** gives you the flexibility to come up with a specific and personal plan
- **I am not sure which tool I prefer**

Allocation method: Random and preference not assessed (Arm A2)

[No additional text]

Allocation method: Screened (Arm B)

We will use the answers you've just given to choose an action plan tool that is best for you.

Allocation method: Choice (Arm C)
We have two tools that can help you come up with a plan. Click on the tool you would like to use.

[then presented with the same options as in Arm A1 (random and preference assessed)
[if participant clicks 'I am not sure which tool I want']:
Here is some more information about the tools. If you are still unsure which one you want to use, we will choose one for you.

- **Smart snacking 101:** gives ideas about ways that people often eat less snacks, so that you can come up with your own simple plan
- **Smart snacking Pro:** gives you the flexibility to come up with a specific and personal plan
- **I am not sure which tool I want**

Action plan: literacy-sensitive

Smart snacking

We want you to plan how you will change your unhealthy snacking behavior each day because forming plans has been shown to improve snacking habits.

Step 1: Snack moments

Sometimes we snack because we are hungry, but there are lots of other reasons too.

Below is a list of '**snack moments**.' These are times when people tend to choose unhealthy snacks or eat too much.

Choose **3** snack moments from the list that happened to you the **most often** in the **last week.**

I often eat unhealthy snacks when…

I am happy	I am bored	I am busy or stressed
I start with one piece but then keep eating	I have a craving	I am drinking alcohol
The snack is right in front of me	I have arrived home	I am drinking tea or coffee
Someone offers me a snack	I want to reward myself	People around me are eating
I am in front of a TV or computer	It is part of a celebration or special event	I am sad

Step 2: Your 'key' snack moment

Below are your top 3 snack moments.

Some snack moments will be more important than others. Choose the **1** that you would be **happiest** to change.

I really want to change my habit of snacking when…

I am bored	I am tired	I am sad

Step 3: Make a plan

Great! Your key snack moment is:
Eating unhealthy snacks when **I am bored.**

The last step is to come up with a plan! **Choose the solution that you think will work best for you.**

Drag it into the space on the right.

Go for a walk	If I want a snack because I am bored, I will…
Listen to music	
Take a break	
Chat to someone for 5 minutes	
Drink tea	
Do a chore or task	
Eat a smaller amount	
Drink a large glass of water	
Eat a piece of fruit	
Take the food out of the packet and put it on a plate	
Eat fresh vegetables and dip	

Step 3: Your plan is almost ready!

Here is your plan so far:

If I want a snack because I am bored [with image]	•	Then I will listen to music [with image]

[Participants are asked to imagine and reflect on the difficulty of carrying out the plan, example below]

Imagine how you plan might feel. **When do you feel bored?**
Sometimes you might eat unhealthy snacks while you:

- Wait for a friend
- Take a long train or bus trip
- Watch TV
- Do jobs or chores you do not like

If this happens, and you want an unhealthy snack, do you think you could listen to music instead?

How hard do you think it will be to do this plan for the next month?
(Very easy to very hard, 0-10 slider)

[For solutions that involve eating fruit instead, we warned that they would need to have this fruit available in advance.]

[if the score was ≥ 7]
Your score shows that this plan may be hard for you.
Let's make a plan that is a bit easier!
[Participants selects a new solution as in Step 3 above]
How hard do you think it will be to do this plan for the next month?
(Very easy to very hard, 0-10 slider)

Your smart snacking plan

Well done! Try to remember this plan for the **next month.** Say it **3 times** to yourself. You can also make a copy of your plan at the end of this survey.

If I want a snack because I am bored [with image]	•	Then I will listen to music [with image]

Action plan: Standard

Smart snacking

Your healthy snacking plan
We want you to plan how you will change your unhealthy snacking behavior each day because forming plans has been show to improve snacking habits.

You are free to choose how you do this but we want your to formulate your plans in as much details as possible.

Pay attention to the **situations** in which you will implement (carry out) these plans. Focus on situations when you are not hungry but find yourself snacking

Please enter your **situations** and your **plan** below

Situations in which I will carry out my plan:

My plan:

How hard do you think it will be to do this plan for the next month?
(Very easy to very hard, 0-10 slider)

Your healthy snacking plan

Well done! Try to remember this plan for the **next month**. Say it a few times to yourself. You can also make a copy of your plan at the end of the survey.

Snacking situations: This is an example response. Participants can list a situation here.

Snacking plan: This is an example response. Participants can write a plan here.

Great work! Now you have a plan to try out for the next month.

[All participants]

We would like to ask a few more questions before you finish:

Difficulty using the tool

How hard was the tool to use?

Not at all hard	A little hard	Somewhat hard	Very hard	Extremely hard

Intentions to reduce unhealthy snacking (immediately post-intervention)

What do you think about snacking? **Over the next month...**

	Strongly disagree	Disagree	Disagree a bit	Neither agree nor disagree	Agree a bit	Agree	Strongly agree
I want to eat fewer (less) unhealthy snacks	☐	☐	☐	☐	☐	☐	☐
I plan to eat fewer unhealthy snacks	☐	☐	☐	☐	☐	☐	☐
I intend (plan) to eat fewer unhealthy snacks	☐	☐	☐	☐	☐	☐	☐

End of baseline survey

[Participants are presented with another copy of their plan (regardless of planning tool) and asked to write it down, save as a PDF or take a screenshot. Participants are asked to click a button that says "Yes, I have a copy of my plan!"]

That's it! Good luck with your smart snacking plan!

Thank you for completing this survey.
We will send you 3 reminders over the next few weeks to help you remember your plan.

Then we will check in with you in **one month** to see how you are going.

Click the 'next' button to submit your responses.

Follow-up survey
Follow-up survey introduction

Smart snacking
Snacks are important because they keep us going until the next meal. Sometimes though, we eat too many, or choose snacks that are unhealthy.

Even though each snack is usually small, over time the snacks add up. This can make us gain weight.

Smart snacking means choosing nutritious, healthy snacks that give you energy until the next meal.

Which snacks are healthy?
Healthy snacks are low in kilojoules, fat, salt and sugars. These include fresh fruit, vegetables with dup, small amounts of dried fruit or nuts, yoghurt, coffee made with low fat milk, raisin toast, rice cracks and corn things.

Which snacks are unhealthy?
Unhealthy snacks are high in kilojoules, fat, salt and sugars. These include biscuits, cheese crackers, cakes, muffins, pastries, chocolate, lollies, potato chips, hot chips, French fries, some muesli bars and large coffees made with full cream milk.

Before we get started on smart snacking, we'd like to know a little more about your snacking habits.

Place your mouse over any words that have a dotted line to see what they mean! Try it out on the words above.

[Mouse-over words shown in brackets below]

Habit strength for unhealthy snacking
Think about your snacking habits in the **last month**. Choose a button for each of the questions below.

	Strongly disagree	Disagree	Disagree a bit	Neither agree nor disagree	Agree a bit	Agree	Strongly agree
1. I eat unhealthy snacks frequently(all the time)	☐	☐	☐	☐	☐	☐	☐
2. I eat unhealthy snacks automatically(without thinking)	☐	☐	☐	☐	☐	☐	☐
3. I eat unhealthy snacks without having to consciously remember (When I eat unhealthy snacks I have not made an active decision) to eat them.	☐	☐	☐	☐	☐	☐	☐

4. I feel weird if I do not eat unhealthy snacks	☐	☐	☐	☐	☐	☐	☐
5. I eat unhealthy snacks without thinking	☐	☐	☐	☐	☐	☐	☐
6. It would require effort not to eat unhealthy snacks	☐	☐	☐	☐	☐	☐	☐
7. Unhealthy snacks belong to are part of my routine(what I usually do)	☐	☐	☐	☐	☐	☐	☐
8. I start eating unhealthy snacks before I realise I'm doing it	☐	☐	☐	☐	☐	☐	☐
9. I would find it hard not to eat unhealthy snacks	☐	☐	☐	☐	☐	☐	☐
10. I don't need to think about eating unhealthy snacks)	☐	☐	☐	☐	☐	☐	☐
11. Unheathy snacking is typical normal for me	☐	☐	☐	☐	☐	☐	☐
12. I've been eating unhealthy snacks for a long time	☐	☐	☐	☐	☐	☐	☐

Extend of perceived unhealthy snacking

Think about your snacking habits in the **last week**. To what extent (How true is it that your snacks this week were) have your snacks this week been:

	Not at all					Very much		
	0	1	2	3	4	5	6	7
**healthy** (for example: apple, banana, or dried fruit)								
**unhealthy** (for example: chocolate, crisps or cake)								

Snacking scores (main outcome)

How often do you usually eat oven baked potato gems/chips/hashbrowns, hot chips / French fries, wedges or fried potatoes?

Each day	Each week	Each month	I don't eat this

In totally, how many serves of potato gems/chips/hashbrowns, hot chips / French fries, wedges or fried potatoes do you usually eat in the timeframe selected above?

1 serve =

- 12 fried hot chips
- 1 cup (60g) potato gems / hashbrowns, or wedges

How many servings (0 to 10)

The same questions will also be asked for the following foods:

- savoury snacks such as crisps, pretzels or plain/flavoured crackers?
  - 1 serve = 1/2 snack size packet of crisps 1 handful (30g) of salty crackers or pretzels
- sweet biscuits/cakes/ buns/ muffins/ doughnuts? Include both home-made and bought.
  - 1 serve = 2-3 (35g) sweet biscuits / 1 donut / 1 slice (40g) of plain cake or sweet bun / 1 small muffin
- savoury pastries? This includes pies, pasties, sausage rolls, Kransky Dogs and frankfurters wrapped in pastry.
  - 1 serve = 1/4 (60g) commerical meat pies or pastie / 1 party size pie or sausage roll
- snack type bars? This includes muesli bars, fruit bars and breakfast cereal bars. *(1 bar = 1 serve)*
- chocolate or lollies? Include all types of chocolate and both hard and soft lollies.
  - 1 serve = ½ chocolate bar /4 pieces of chocolate (25g) /5-6 (40g) lollies
- ice-cream or ice-blocks? This includes ice-blocks, ice-cream in a bowl or ice-creams on a stick.
  - 1 serve = 2 scoops (60g) ice-cream / 1 stick ice-cream or ice-block
- soft drink, cordial or sports drinks? Include all drinks with **added sugar** such as soft drinks, cordials, fruit drinks, vitamin waters, energy and sports drinks.
  - 1 serve = 1 can (375mL of soft drink) / 1 bottle of sports drink / 375mL of cordial or fruit drink
- How often do you usually eat meat products? Include sausages, frankfurters, devon, fritz, ham, salami, hot dogs, hamburgers and chicken nuggets
  - 1 serve = 2 slices (55g) processed meat such as ham, salami, devon or fritz / 2 thin of 1 ½ thick (60g) sausages, frankfurters or hot dogs / 1 hamburger pattie / 3 (60g) chicken nuggets

Intentions to reduce unhealthy snacking

What do you think about snacking?

Over the next month...

	Strongly disagree	Disagree	Disagree a bit	Neither agree nor disagree	Agree a bit	Agree	Strongly agree
1. I want to eat fewer (less) unhealthy snacks	☐	☐	☐	☐	☐	☐	☐
2. I plan to eat fewer unhealthy snacks	☐	☐	☐	☐	☐	☐	☐
3. I intend (plan) to eat fewer unhealthy snacks	☐	☐	☐	☐	☐	☐	☐

Action control for reducing unhealthy snacking

How did you go with your snacking plan?

During the last month…

	Strongly disagree	Disagree	Disagree a bit	Neither agree nor disagree	Agree a bit	Agree	Strongly agree

1. I constantly monitored (often watched) how many unhealthy snacks I ate to make sure it was not too much	☐	☐	☐	☐	☐	☐	☐
2. I watched carefully to make sure I was following my plan to eat less unhealthy snacks	☐	☐	☐	☐	☐	☐	☐
3. I often thought about my plan to eat less unhealthy snacks	☐	☐	☐	☐	☐	☐	☐
4. I was always aware of my plan to eat less unhealthy snacks	☐	☐	☐	☐	☐	☐	☐
5. I really tried to eat less unhealthy snacks	☐	☐	☐	☐	☐	☐	☐
6. I tried my best to act in accordance with my standards (stick with my personal goals)	☐	☐	☐	☐	☐	☐	☐

Post-intervention preference for action plans

[Participants are reminded which action plan they used and are shown screenshots of the other action plan. Example given below]

In this study you created your plan using [name and picture of plan].

Below are screenshots from another snacking tool called [name and picture of the other plan]

[Screenshots of plan shown using a slider HTML feature]
If you were given a choice, which would you prefer to use next time?

- **Smart snacking 101:** helps you create a simple plan using common ways to eat less snacks
- **Smart snacking Pro:** helps you create a tailored plan that suits your specific situation
- **I am not sure which tool I prefer**

[If they select ‘I am not sure which tool I would prefer’]
Here is some more information about the tools.

- **Smart snacking 101:** gives ideas about ways that people often eat less snacks, so that you can come up with your own simple plan
- **Smart snacking Pro:** gives you the flexibility to come up with a specific and personal plan
- **I am not sure which tool I prefer**

Difficulty following the plan

How hard was it to follow your plan?
[Very easy to very hard , 0 to 10]

Qualitative feedback questions / end material

Think about the times when it was hard to follow your plan. What happened?

____

[If the participant had used the health-literate action plan at baseline]

The **snack moment** was the situation when you ate unhealthy snacks. Examples included: when you are bored, have a craving, or are in front of a screen.

The **Solution** was the action you took instead of snacking.
Examples included: drinking water, eating fruit or going for a walk.

Were there any **snack moments** or **solutions** that were missing from the list?

Snack moments: _________________________________
Solutions: ______________________________________

[All participants]
Do you have any other feedback? ________________________________

That is the end of the survey, thank you for your participation.
If you would like more information about the study, please click here (opens in a new window).
If you would like to receive a copy of the results of this study, please email the study office at julie.ayre@sydney.edu.au
Please click the red button below to submit the survey.
