# Supplementary material for: Effects of health literacy, screening, and participant choice on action plans for reducing unhealthy snacking in Australia: A randomised controlled trial
Source: PLoS Med. 2020 Nov 3;17(11):e1003409. doi: 10.1371/journal.pmed.1003409 (PMC7608866; doi:10.1371/journal.pmed.1003409)
Supplement: S1 Text — (PDF) [file pmed.1003409.s003.pdf]

S1 Text: Protocol, CONSORT and TIDieR Checklists

This supplementary file contains the following:

Table A. Protocol (including links to published protocol and ANZCTR trial registration)
Table B. CONSORT 2010 Checklist of information to include when reporting a randomised trial
Table C. The TIDieR (Template for Intervention Description and Replication) Checklist

Table A. Protocol (including links to published protocol and ANZCTR trial registration)

**Link to BMJ Open published protocol**	https://bmjopen.bmj.com/content/9/5/e028544.info
**Link to ANZCTR trial registration**	https://www.anzctr.org.au/Trial/Registration/TrialReview.aspx?id=375665
**Introduction**	Health literacy describes the cognitive and social skills that enable individuals to access, understand and act on health information.[1] Low health literacy is increasingly recognised as an important contributor to health inequality and is associated with increased hospitalisation, mortality, prevalence of chronic disease and risk factors for health conditions.[2] Low health literacy is common worldwide, with estimates ranging from 36% to 60% of the population in Australia, Europe and the USA.[3–5]<br>Current approaches to address health literacy issues have focused on providing all consumers, regardless of their health literacy level, with health information that is easy to process and understand.[2] 6 While this 'universal precautions approach' has been shown to improve health knowledge, it is less clear whether it is effective for improving complex behaviours such as healthy eating and increased physical activity.[2 7 8] This may reflect the fact that health literacy guidelines and interventions place relatively little emphasis on strategies that promote action, such as planning, self-monitoring or problem solving.[9–13] These kinds of strategies are increasingly recognised as key components of lifestyle interventions[14]; furthermore, there has been little research investigating how they could be adapted for audiences with lower health literacy.<br>A recent randomised controlled trial (RCT) has investigated the effects of literacy-sensitive design on action plans to reduce unhealthy snacking behaviour.[15] This design incorporated health literacy strategies (eg, simple language) and separated the planning process into distinct steps to reduce cognitive demands. This 'literacy-sensitive' action plan was compared with action plan instructions that have been used in samples of the general population.[16] The results from this study suggested that people with lower health literacy reported consuming fewer unhealthy snacks at follow-up when they had used the literacy-sensitive action plan, whereas people with higher health literacy reported consuming fewer unhealthy snacks using the 'standard' action plan.[15]<br>The current study will build on these findings by evaluating the most effective way to determine the best action plan for participants. One obvious approach is to allocate an action plan based on a participant's health literacy score. Alternatively, participants could be asked to select the plan they would prefer to follow. Although allowing the participant to choose their plan might less accurately match a participant to an action plan that meets their health literacy needs, there are some potential additional advantages to this approach. For example, participants would be able to factor in other relevant aspects (such as their motivation to engage with the planning process),[17] and presenting participants with different options may also encourage greater satisfaction with the intervention.[18] This is further supported by evidence that the effects of interventions in RCTs may be increased when participants receive their preferred intervention.[19]

	This study has three key aims. First, this study aims to evaluate the impact of health literacy and a literacy-sensitive action plan on unhealthy snacking in a sample of people with type 2 diabetes and/or overweight or obese body mass index (BMI). In doing so, this study aims to replicate previous findings in a clinical sample.[15] Unhealthy snacking in this study includes consumption of discretionary foods as described in the Australian Guidelines to Healthy Eating.[20] For the purposes of this study, this included sugar-sweetened beverages and excluded alcoholic beverages.<br><br>The second aim is to evaluate how the method of allocation to either intervention (literacy-sensitive or standard action plan) affects the overall effectiveness of the intervention. Three methods of allocation will be evaluated: (1) random allocation to an action plan; (2) allocation to an action plan based on individual health literacy or (3) allowing participants to choose which action plan they use. This study will employ a two-stage randomisation (Rucker) design.21 This design allows estimation of the effects of each action plan on outcomes (the treatment effect), independent of the effects of a person receiving their preferred treatment (preference effect) and the effects of self-selection (selection effect). This is the only preference trial design that allows this delineation of all these effects.[22]<br><br>The third aim is to evaluate whether assessment of participant preference for an intervention prior to random allocation influences the effectiveness of the intervention. This addresses an unanswered question in research on the effects of treatment preference on study outcomes, that is, does assessment of preference introduce a methodological artefact by increasing the salience of discrepancies between an individual's preferred and received treatments and in doing so, have a negative effect on intervention outcomes[22] ?
**Hypotheses**	We hypothesise that:<br>1. A literacy-sensitive action plan will be more effective at reducing unhealthy snacking for participants with lower health literacy, whereas the standard action plan will be more effective for participants with higher health literacy.<br>2. The intervention will be more effective at reducing unhealthy snacking for participants who are allocated an action plan using the health literacy screening tool compared with those who are asked to select their preferred action plan. Both of these allocation methods will be more effective than random allocation to an action plan.<br>3. Assessing preference will negatively impact plan effectiveness, an effect which will be greater for those who are randomised to the plan which is discordant with their preference.
**Methods and analysis**	**Study design**<br>The design is a three parallel-arm online RCT to test the effect of health literacy, type of action plan and method of allocation to an action plan on self-reported unhealthy snacking behaviour. The three methods of allocation are (1) random, (2) use of a health literacy screening tool to allocate participants one of the two action plans ('screened' arm) and (3) participant choice of action plan ('choice' arm). This study will also evaluate whether the process of assessing preference for a particular action plan prior to randomisation will have an impact on subsequent self-reported snacking behaviour. A schematic representation of the study design is shown in figure 1 of the open-access protocol (https://bmjopen.bmj.com/content/9/5/e028544.info).<br>**Participants and recruitment**<br>The proposed study will seek to recruit 2352 Australian participants with type 2 diabetes. Participants will be recruited through an online market research company,

	Dynata, which has a pool of approximately 10 000 Australians with type 2 diabetes. This sample will be supplemented with additional participants from Dynata who have self-reported height and weight corresponding to an overweight or obese BMI (ie, BMI ≥25 kg/m2). Participants will be eligible to participate if they are registered with Dynata's Australian registry, are over 18 years of age (adult population) and self-report that they have type 2 diabetes or self-report a height and weight that correspond to overweight or obese BMI. Participants will be excluded if they do not speak English. Participants will not be excluded on the basis of their snacking behaviour. Recruitment commenced on 14 February 2019.<br>Participants who click the link received from Dynata will be presented with a brief introduction to the study and a link to the participant information sheet (see online supplementary file 1 of the open access protocol (https://bmjopen.bmj.com/content/9/5/e028544.info)). Informed consent will be indicated by completion of the online survey, as outlined in the participant information sheet. On the next page, participants then begin the baseline survey.<br>**Patient and public involvement**<br>Patients and public were not directly involved in development of the research question, however, consumer health representatives living with type 2 diabetes were consulted for feedback at multiple stages of intervention and study development, including: intervention instructions, the appropriateness of the literacy-sensitive plans and the ease with which feedback at follow-up could be communicated. Participants in the trial are able to indicate if they are interested in receiving a lay summary of the study results which will be disseminated through email by Dynata (further maintaining participants anonymity). We will also assess participant burden and acceptability using the pilot data. Lastly, participants will be able to provide feedback on the perceived burden of the intervention using free text fields during the follow-up survey.<br>**Participant allocation**<br>After completing baseline measures, participants will be randomised to one of three allocation methods using the 'Survey Flow' and 'Randomiser' functions included in the survey platform (Qualtrics) (figure 1). The 'Randomiser' is based on the Mersenne Twister, a pseudorandom number generator. This allocation method will determine how the planning tool (either literacy-sensitive or standard) is assigned to the participant. Participants in the random arm will be unaware of their allocation method. At baseline, only the participants in the choice arm and those who are randomised to assess prior preference in the random arm will be aware of the two different tool versions. Participants in the screened arm will be aware that there is more than one tool available.<br><br>**Allocation method**<br>Random (arm A): Participants randomised to the 'random' arm will be further randomised to either (1) assess their prior preferences (ie, their preferred action plan; arm A1), and then randomised to the standard or literacy-sensitive action plan or (2) randomised to the standard or literacy-sensitive action plan without assessment of prior preferences (arm A2; figure 1). Prior preference will be measured using the same format as in the 'participant choice' arm (arm C) with additional text stating that participants may not receive their preferred tool.<br>Screened (arm B): Allocation is based on assessment of health literacy using the Newest Vital Sign (NVS) measure.23 The literacy-sensitive action plan will be allocated to those scoring less than 4 (indicative of inadequate health literacy), and the standard action plan will be allocated to the remaining participants. Participants will be told that, based on their responses, the researchers have selected an action plan tool that is most suitable for them.<br>Choice (arm C): Participants will be provided with a brief description of the action plans and select the plan they want to use. The literacy-sensitive intervention is described in simple language whereas the standard intervention is described using more complex

	words (eg, 'tailored plan that suits your specific situation' vs 'simple plan using common ways to eat less snacks'). It is anticipated that the difference in language complexity will help participants select the most appropriate tool for their health literacy level by giving an indirect indication of the relative level of cognitive effort required, in addition to the explicit descriptions which describe the relatively greater cognitive effort required in the standard plan. Order of presentation of the two plans is randomised. In the first instance, participants have the option of selecting 'Unsure' to allow for undecided participants. Participants will then be presented with an alternative description of the study and asked again to make a choice. Participants will be informed that if they select 'unsure' again, the researchers will select a plan for them. In doing so, participants will be randomised to an action plan as per Rucker protocol.[21]<br><br>**Action plan interventions**<br>Either a literacy-sensitive or standard action plan will be allocated to participants. The text used in each tool is presented below:<br><br>**Literacy-sensitive action plan ('smart snacking 101 (basic)')**<br>This commences with the text: 'We want you to plan how you will change your unhealthy snacking behaviour each day because forming plans has been shown to improve snacking habits'. The intervention consists of four steps that guide the participant through the process of developing an appropriate plan (also shown in figure 2 of the open access protocol (https://bmjopen.bmj.com/content/9/5/e028544.info)). The steps are also outlined below:<br>Step 1: Sometimes we snack because we are hungry, but there are lots of other reasons. Think about your snacks in the last week. Below is a list of 'snack moments.' These are times when people tend to choose unhealthy snacks or eat too much. Choose three snack moments from the list that happened to you the most often in the last week. (Participants selects from the list of snack moments).<br>Step 2: Below are your top three snack moments. Some snack moments will be more important than others. Choose the one that you would be happiest to change. (Participant chooses from three previously selected snack moments).<br><br>Step 3: Great! Your most important snack moment was snacking because you are (example snack moment: bored). The last step is to come up with a plan! Choose the solution that you think will work best for you. Drag it into the space on the right. (Participants selects from the list of solutions).<br>Step 4: Imagine how your plan might feel (examples of scenarios when this might happen). The final step is to make sure the plan is realistic. How hard do you think it will be to do this plan for the next month (Slider options range from very easy [1] to very hard [10]. If the participant selects a number greater than or equal to 7 they will be prompted to revise the plan).<br><br>**Standard action plan ('smart snacking pro (advanced)')**<br>Participants receive the following instructions: 'We want you to plan how you will change your unhealthy snacking behaviour each day, because forming plans has been shown to improve snacking habits. You are free to choose how you do this but we want you to formulate your plans in as much detail as possible. Pay attention to the situations in which you will implement (carry out) these plans. Focus on situations when you are not hungry but find yourself snacking. Please enter your situations and your plans below.'<br><br>After completing either action plan, participants will be presented with their plan for a final time and instructed to write down, take a screenshot or make a copy of it. Participants will also be asked to confirm that they have a copy of the plan. We have

	incorporated behaviour change techniques into each intervention. These are described in detail in the online supplementary file 2 of the open access protocol (https://bmjopen.bmj.com/content/9/5/e028544.info).<br><br>**Baseline and follow-up surveys**<br>At baseline, participants will complete demographic questions and measures of health literacy. They will then receive information about general reasons for reducing unhealthy snacking (such as avoiding weight gain), reasons for 'smart snacking' (ie, choosing healthy snacks), definitions of healthy and unhealthy snacks (low and high in: kilojoules, fat, salt and sugars, respectively), and examples from each category. Participants then complete measures of snacking behaviour, habit strength (concerning consumption of unhealthy snacks) and intentions to reduce unhealthy snacking. Intention to reduce unhealthy snacking will be measured again, immediately after creating the plan. Participants will be emailed a reminder of their personal plan at baseline (within the first week), and before then end of the second and third weeks to increase compliance and retention. Consistent with our previous study, in which a change in snacking scores was detected as a result of the intervention after 4 weeks,[15] participants in this study will also complete a follow-up survey after 4 weeks. In addition, the 4-week period was selected as it is a tangible time period over which participants can recall their behaviour, and the instrument for the primary outcome has been validated for recall over the previous month.[20] The follow-up survey consists of the same description of reasons to reduce unhealthy snacking and definition, followed by measures of snacking behaviour, habit strength and intention. Action control will also be measured in the follow-up survey.<br><br>**Measures**<br>*Screening measures*<br>Prior to beginning the survey, participants will be asked to indicate whether they have type 2 diabetes, and to provide their height (cm or feet and inches) and weight (kg).<br>*Demographic measures*<br>Participants will be asked to complete questions about their age, employment status, highest level of educational attainment, and, if they report having diabetes, years since diagnosis and whether or not they use insulin.<br>*Health literacy*<br>Health literacy will be measured using the NVS,[23] a 6-item measure of functional health literacy, and a single-item literacy screener.[24] NVS scores of 0–1 indicate a high likelihood of limited healthy literacy, scores of 2–3 indicate the possibility of limited health literacy and scores of 4–6 indicate adequate health literacy.<br>*Need for cognition*<br>Need for cognition describes the extent that an individual engages in and enjoys cognitively effortful activities (ie, activities that require a lot of thinking).[25] Three items on a 7-point Likert scale (strongly disagree to strongly agree) will assess the participant's need for cognition.[26]<br><br>**Primary outcome: Snack scores (previous month)**<br>Snacking scores will be measured using a validated 7-item measure based on a diet score developed by the Commonwealth Scientific and Industrial Research Organisation.[20] Items are drawn from the 'discretionary foods' category which the Australian Guidelines to Healthy Eating define as foods 'not considered necessary for a healthy diet'. Alcohol was excluded from the assessment in this study as the focus is on 'snacks.' Participants answer how many serves of unhealthy snacks they ate in the past month. Participants can answer according to the number of serves per day, week or month. Average weekly serves of unhealthy snacks will be calculated from these scores. Although electronic diaries might overcome some limitations of once-off measures of snacking behaviour

	such as that described above, electronic diaries were unavailable at the time of study development due to the budget and technical constraints. This approach was chosen also reduces participant burden and minimises the risk of missing data.<br><br>## Secondary outcomes<br>*Perceived unhealthy/healthy snacking (previous week)*<br>Two items, each on a 7-point Likert scale will assess perceived extent of healthy and unhealthy snacking in the previous week, respectively. Healthy snacks are described to participants as those that are low in kilojoules, fat, salt and sugars. Unhealthy snacks are described as high in kilojoules, fat, salt and sugars. Examples of healthy and unhealthy snacks are provided.<br>*Intention, habit strength and action control*<br>The measure of intention consists of 3 items that ask about the participant's intention to reduce unhealthy snacking.[27 28] Habit strength will be assessed using the 12-item self-report habit strength index,[29] and action control will be assessed using a 6-item measure.[30] Responses to each item are recorded on 7-point Likert scales (strongly disagree to strongly agree).<br>*Difficulty using the planning tool*<br>A single item will ask participants to rate how hard it was to use the planning tool (1=not at all hard, 5=extremely hard).<br>*Preferred action plan at follow-up*<br>At follow-up, participants will be reminded of the name and logo used for their plan. Participants will be shown an image slider that contains screenshots from the other action plan. Participants will then be asked: 'If you were given the choice, which action plan would you prefer to use next time?'<br><br>## Sample size<br>The proposed study will seek to analyse a sample of 2000 Australian participants at follow-up with high BMI (overweight/obese) and/or type 2 diabetes. At baseline participants will be randomised at a ratio of 2:1:1 to each allocation method arm, such that there are a total of 1000 participants in the random arm (who will then be evenly randomised to assess or not assess their preference for a particular action plan before they are ultimately randomised to an action plan), and 500 in the two remaining allocation method arms (the screened and choice arms). With a two-sided alpha of 0.05 and power of 80%, a sample of this size will allow us to detect a small main effect of f=0.08 in a univariate analysis of variance (ANOVA) comparing the three allocation method arms and in a univariate ANOVA comparing the two prior-preference assessment arms; this corresponds to a minimum pairwise difference between the two most extreme mean values of approximately 0.18 SD. Estimates of effect size are based on the outcome of previous work exploring the effects of action plans on unhealthy snacking.[15]<br><br>Based on our previous studies recruiting through this provider, we anticipate an attrition rate no greater than 15% by 1-month follow-up. Therefore, we estimate a total of 2352 should be recruited at baseline to ensure sufficient sample size for analysis.<br><br>A sample of this size will also ensure that there is at least 80% power for secondary analysis conducted to estimate treatment and preference effects[20 31] with a treatment effect between the two interventions (literacy-sensitive and standard planning tools) as small as 0.25 SD (estimated as five snacks per month based on the previous findings[15]), and a preference effect, comparing those who received their preferred tool to those who did not, as small as 0.35 SD (approximately seven snacks per month). This assumes that approximately equal proportions of participants will choose the literacy-sensitive and standard planning tools in the choice arm, and that there will be approximately

	equal proportions of participants allocated to each of these interventions in the screened arm.<br><br>**Piloting**<br>The intervention will be piloted with 200 participants to check that approximately equal numbers of participants allocated to the choice condition select each of the interventions. If required, sample size estimates will be adjusted to ensure that the study is sufficiently powered. Piloting will also allow us to assess participant burden and intervention acceptability.<br><br>**Analysis**<br>Follow-up outcome measures for participants who do not complete the follow-up survey will be estimated for worst-case (no change in snacking score) and best-case scenarios (average change in snack score). These estimates will be incorporated as an intention-to-treat analysis. Baseline characteristics of completers and non-completers will be compared to assess bias and generalisability.<br><br>**Confirmatory analysis**<br>A confirmatory analysis will replicate the analysis previously reported[15] to examine if the treatment effect is modified by health literacy. Multiple linear regression models including an intervention group × health literacy (NVS score) interaction term will be used to predict follow-up snacking scores and perceived difficulty using the plan. Important correlates of health literacy (age, level of education, language spoken at home),[2] diabetes status and baseline snacking will be controlled for in the model. NVS scores will be examined both continuously and categorically (ie, inadequate vs adequate health literacy).<br><br>**Assessment of prior preference analysis**<br>For participants in the random arm of the study, participants whose action plan preference was assessed prior to randomisation to an intervention will be compared with those whose preference was not assessed. Multiple linear regression (controlling for health literacy, age, level of education, language spoken at home and baseline snacking) will evaluate the effect of preference assessment on unhealthy snacking behaviour. For participants who provided a preference, an additional multiple linear regression will evaluate the effect of participants receiving their preferred intervention compared with those not receiving their preferred intervention on unhealthy snacking behaviour.<br><br>**Analysis of effects of allocation method**<br>The primary analysis will use regression to test for a difference in self-reported unhealthy snacking between the three randomised arms (random, screened and choice) while adjusting for any effect of diabetes status. An adjusted model will also be constructed to allow for any baseline imbalances in the potential confounders including age, English as a second language, level of education. The analysis will be repeated on the secondary outcomes of perceived unhealthy snacking in the previous week, snacks consumed the previous day, difficulty using the planning tool, action control and habit strength with similar adjustment for diabetes status and any baseline imbalances. A subanalysis will also be conducted on participants with and without type 2 diabetes.<br><br>An appropriate analysis that uses the available preference information will be used to estimate treatment, preference and selection effects for the primary outcome.[32] The treatment effect compares the efficacy of the health literate action plan with the standard action plan in the random arm. The preference effect and selection effects are

	estimated using the random and choice arms. The preference effect measures the difference in self-reported unhealthy snacking for those who received their preferred action plan compared with those who did not receive their preferred action plan. The selection effect measures the difference between those who would select the literacy-sensitive intervention with those who would select the standard intervention regardless of which intervention they received. Secondary analyses will adjust for diabetes status, and baseline imbalances in potential confounders including age, English as a second language, level of education. Data from participants in the screened arm will also be analysed in this manner, producing estimates analogous to the preference and selection effects.<br>These effects will also be estimated for the secondary outcomes of perceived unhealthy snacking in the previous week, snacks consumed the previous day, difficulty using the planning tool, action control and habit strength with similar adjustment for diabetes status and any baseline imbalances.<br>Bootstrapping, by taking repeated random samples with replacement, will be used to estimate the difference between the preference effect (estimated using choice arm) and an analogous effect estimated using the screened arm, as well as the difference between the selection effect (estimated using the choice arm) and an analogous effect estimated using the screened arm. Bootstrapping will also be used to estimate the confidence intervals for non-continuous outcome measures. The bootstrapping is necessary, as the variances for the estimated differences and non-continuous outcomes have not been derived previously.[33]<br>**Additional analysis**<br>Two researchers will also independently code standard action plans to indicate the extent that participants followed standard action plan instructions (ie, provided at least one 'situation' and one 'plan') and the extent that plans differed from the predetermined options presented in the literacy-sensitive action plans (eg, situations or solutions that did not correspond to options listed in the literacy-sensitive action plan, or strategies that fell outside of the 'If-then' construction. The latter may include, for example, making sure healthy snacks are prepared in advance, or refraining from buying certain foods at the supermarket). Coders will be blind to the health literacy level of participants. Any disagreements will be systematically resolved through discussion. Results from this content analysis will also inform a secondary analysis of the impact of allocation method and action plan on snacking scores.<br>**Amendments to analysis plan after data collection**<br>• Snacks consumed the previous day is mentioned in the section 'Analysis of the effects of allocation method.' This variable was in fact removed prior to recruitment to minimise participant burden.<br>• Habit strength was included as a covariate due to baseline differences.
**Data management**	Deidentified data will be captured electronically on the secure Qualtrics server. Data will be stored securely in deidentified form, with a unique participant ID to link responses from the baseline and follow-up surveys. In all forms of dissemination, only deidentified data will be presented as group means and differences to maintain the anonymity of participants. In line with National Health and Medical Research Council recommendations, and University of Sydney policies, all data will be kept for a minimum of 5 years.
**Ethics and dissemination**	The interventions used in this study are practical; they can be easily incorporated into existing self-management practices, particularly with the use of online technologies, and are low in cost. Ascertaining an effective way to tailor planning tools to health literacy level (ie, allowing the participant to choose by employing a screening tool) will provide valuable information for implementation in apps that target people with varying health

	literacy levels. Findings from this study will be disseminated through peer-reviewed publication and national and international conference presentations. Findings will also be disseminated through community and research partnerships with groups such as Western Sydney Diabetes.
**Acknowledgements**	We would like to thank our type 2 diabetes consumer health representatives Edward Hartley and Mike Font for their feedback and input into the design of this study. We would also like to acknowledge the CSIRO for their permission to use items from the discretionary foods components of the CSIRO diet score.
**References**	1. Nutbeam D. Health literacy as a public health goal: a challenge for contemporary health education and communication strategies into the 21st century. Health Promot Int2000;15:259–67.doi:10.1093/heapro/15.3.259<br>2. Berkman ND, Sheridan SL, Donahue KE, et al. 2011. Health literacy interventions and outcomes: An updated systematic reviewEvidence report/technology assessment. 199: 1–941.<br>3. Australian Bureau of Statistics. Health Literacy, Australia cat. no.4233.0. Canberra: Australian Burea of Statistics, 2006.<br>4. Kutner M, Greenburg E, Jin Y, et al. The health literacy of america's adults: results from the 2003 national assessment of adult literacy. (NCES 2006-483). Washington, DC: National Center for Education Statistics, 2006.<br>5. Kickbush I, Pelikan JM, Apfel F, Tsouros ADWorld Health Organization. In: Kickbush I, Pelikan JM, Apfel F, Tsouros AD, et al. Health literacy: the solid facts: World Health Organization, 2013.<br>6. Meppelink CS, Smit EG, Diviani N, et al. Health Literacy and Online Health Information Processing: Unraveling the Underlying Mechanisms. J Health Commun2016;21:109–20.doi:10.1080/10810730.2016.1193920<br>7. Sheridan SL, Halpern DJ, Viera AJ, et al. Interventions for individuals with low health literacy: a systematic review. J Health Commun2011;16 Suppl 3:30–54.doi:10.1080/10810730.2011.604391<br>8. Bailey SC, Brega AG, Crutchfield TM, et al. Update on health literacy and diabetes. Diabetes Educ2014;40:581–604.doi:10.1177/0145721714540220<br>9. Schwarzer R. Modeling health behavior change: how to predict and modify the adoption and maintenance of health behaviors. Appl Psychol2008;57:1–29.doi:10.1111/j.1464-0597.2007.00325.x<br>10. Hagger MS, Chatzisarantis NL. An integrated behavior change model for physical activity. Exerc Sport Sci Rev2014;42:62–9.doi:10.1249/JES.0000000000000008<br>11. Brega A, Barnard J, Mabachi NM, et al. AHRQ health literacy universal precautions toolkit. 2nd Edn. Rockville, MD: Agency for Healthcare Research and Quality, 2015. http://www.ahrq.gov/professionals/quality-patient-safety/quality-resources/tools/literacy-toolkit/healthlittoolkit2.html<br>12. Kim SH, Lee A. Health-literacy-sensitive diabetes self-management interventions: a systematic review and meta-analysis. Worldviews Evid Based Nurs2016;13:324–33.doi:10.1111/wvn.12157<br>13. Schaffler J, Leung K, Tremblay S, et al. The effectiveness of self-management interventions for individuals with low health literacy and/or low income: a descriptive systematic review. J Gen Intern Med2018;33:510–23.doi:10.1007/s11606-017-4265-x<br>14. Michie S, Abraham C, Whittington C, et al. Effective techniques in healthy eating and physical activity interventions: a meta-regression. Health Psychol2009;28:690–701.doi:10.1037/a0016136<br>15. Ayre J, Bonner C, Cvejic E, et al. Randomized trial of planning tools to reduce unhealthy snacking: Implications for health literacy. PLoS One2019;14:e0209863.doi:10.1371/journal.pone.0209863

	16. Armitage CJ. Field experiment of a very brief worksite intervention to improve nutrition among health care workers. J Behav Med2015;38:599–608.doi:10.1007/s10865-015-9634-5<br>17. Smith SG, Curtis LM, Wardle J, et al. Skill set or mind set? Associations between health literacy, patient activation and health. PLoS One2013;8:e74373.doi:10.1371/journal.pone.0074373<br>18. Nguyen MH, Smets EMA, Bol N, et al. How tailoring the mode of information presentation influences younger and older adults' satisfaction with health websites. J Health Commun2018;23:170–80.doi:10.1080/10810730.2017.1421729<br>19. Preference Collaborative Review Group. Patients' preferences within randomised trials: systematic review and patient level meta-analysis. BMJ2008 :337a1864.doi:10.1136/bmj.a1864Abstract/<br>20. Hendrie GA, Baird D, Golley RK, et al. The CSIRO Healthy diet score: an online survey to estimate compliance with the australian dietary guidelines. Nutrients2017;9:47.doi:10.3390/nu9010047<br>21. Rücker G. A two-stage trial design for testing treatment, self-selection and treatment preference effects. Stat Med1989;8:477–85.doi:10.1002/sim.4780080411<br>22. Walter SD, Turner R, Macaskill P, et al. Beyond the treatment effect: evaluating the effects of patient preferences in randomised trials. Stat Methods Med Res2017;26:489–507.doi:10.1177/0962280214550516<br>23. Weiss BD, Mays MZ, Martz W, et al. Quick assessment of literacy in primary care: the newest vital sign. Ann Fam Med2005;3:514–22.doi:10.1370/afm.405Abstract/<br>24. Wallace LS, Rogers ES, Roskos SE, et al. Brief report: screening items to identify patients with limited health literacy skills. J Gen Intern Med2006;21:874–7.doi:10.1111/j.1525-1497.2006.00532.x<br>25. Cacioppo JT, Petty RE. The need for cognition. J Pers Soc Psychol1982;42:116–31.doi:10.1037/0022-3514.42.1.116<br>26. Nikoloudakis IA, Crutzen R, Rebar AL, et al. Can you elaborate on that? Addressing participants' need for cognition in computer-tailored health behavior interventions. Health Psychol Rev2018;12:437–52.doi:10.1080/17437199.2018.1525571<br>27. Verhoeven AAC, Adriaanse MA, de Ridder DTD, et al. Less is more: the effect of multiple implementation intentions targeting unhealthy snacking habits. Eur J Soc Psychol2013;43:344–54.doi:10.1002/ejsp.1963<br>28. Verhoeven AA, Adriaanse MA, de Vet E, et al. Identifying the 'if' for 'if-then' plans: combining implementation intentions with cue-monitoring targeting unhealthy snacking behaviour. Psychol Health2014;29:1476–92.doi:10.1080/08870446.2014.950658<br>29. Verplanken B, Orbell S. Reflections on past behavior: a self-report index of habit strength. J Appl Soc Psychol2003;33:1313–30.doi:10.1111/j.1559-1816.2003.tb01951.x<br>30. Sniehotta FF, Scholz U, Schwarzer R. Bridging the intention–behaviour gap: planning, self-efficacy, and action control in the adoption and maintenance of physical exercise. Psychol Health2005;20:143–60.doi:10.1080/08870440512331317670<br>31. Turner RM, Walter SD, Macaskill P, et al. Sample size and power when designing a randomized trial for the estimation of treatment, selection, and preference effects. Med Decis Making2014;34:711–9.doi:10.1177/0272989X14525264<br>32. McCaffery KJ, Turner R, Macaskill P, et al. Determining the impact of informed choice: separating treatment effects from the effects of choice and selection in randomized trials. Med Decis Making2011;31:229–36.doi:10.1177/0272989X10379919<br>33. Cameron B, Peduzzi P, Esserman D. Extensions to the two-stage randomized trial design for testing treatment, self-selection, and treatment preference effects to binary outcomes. Stat Med2018;37:3147–78.doi:10.1002/sim.7830

Table B. CONSORT 2010 checklist of information to include when reporting a randomised trial*

Section/Topic	Item No	Checklist item	Reported on page No
**Title and abstract**			
	1a	Identification as a randomised trial in the title	Title page
	1b	Structured summary of trial design, methods, results, and conclusions (for specific guidance see CONSORT for abstracts)	Abstract
**Introduction**			
Background and objectives	2a	Scientific background and explanation of rationale	Introduction Paragraphs 1-8
	2b	Specific objectives or hypotheses	Introduction paragraph 9 (final paragraph)
**Methods**			
Trial design	3a	Description of trial design (such as parallel, factorial) including allocation ratio	Methods: study design, Fig 1; Methods: Participant allocation
	3b	Important changes to methods after trial commencement (such as eligibility criteria), with reasons	None, except that mentioned paragraph 1 under section Results: Aim 1 Effects of health literacy and action plan
Participants	4a	Eligibility criteria for participants	Methods: Participants and recruitment
	4b	Settings and locations where the data were collected	Methods: Participants and recruitment
Interventions	5	The interventions for each group with sufficient details to allow replication, including how and when they were actually administered	Methods: Allocation method plus protocol paper[a]
Outcomes	6a	Completely defined pre-specified primary and secondary outcome measures, including how and when they were assessed	Methods: Measures
	6b	Any changes to trial outcomes after the trial commenced, with reasons	NA
Sample size	7a	How sample size was determined	Methods: Sample size
	7b	When applicable, explanation of any interim analyses and stopping guidelines	NA (Analysis methods presented in section Methods: Analysis)
Randomisation:			
Sequence generation	8a	Method used to generate the random allocation sequence	Specified in protocol paper[a]
	8b	Type of randomisation; details of any restriction (such as blocking and block size)	Specified in protocol paper[a]
Allocation concealment mechanism	9	Mechanism used to implement the random allocation sequence (such as sequentially numbered containers), describing any steps taken to conceal the sequence until interventions were assigned	Specified in protocol paper[a]
Implementation	10	Who generated the random allocation sequence, who enrolled participants, and who assigned participants to interventions	Specified in protocol paper[a]

Blinding	11a	If done, who was blinded after assignment to interventions (for example, participants, care providers, those assessing outcomes) and how	see under Methods: Analysis: Additional qualitative analysis of action plan content
	11b	If relevant, description of the similarity of interventions	NA
Statistical methods	12a	Statistical methods used to compare groups for primary and secondary outcomes	Methods: analysis: sections for Aim 1 and Aim 2
	12b	Methods for additional analyses, such as subgroup analyses and adjusted analyses	Methods: analysis section; see also paragraph 4 in the results section for Aim 1: primary outcome; paragraph 2 in results section for Aim 2: primary outcome
**Results**			
Participant flow (a diagram is strongly recommended)	13a	For each group, the numbers of participants who were randomly assigned, received intended treatment, and were analysed for the primary outcome	Fig 1
	13b	For each group, losses and exclusions after randomisation, together with reasons	Fig 1
Recruitment	14a	Dates defining the periods of recruitment and follow-up	Methods: Participants and recruitment; 4-week followup Methods: Baseline and follow-up surveys paragraph 2
	14b	Why the trial ended or was stopped	NA
Baseline data	15	A table showing baseline demographic and clinical characteristics for each group	Table 1
Numbers analysed	16	For each group, number of participants (denominator) included in each analysis and whether the analysis was by original assigned groups	S3 Table S4; Fig 1
Outcomes and estimation	17a	For each primary and secondary outcome, results for each group, and the estimated effect size and its precision (such as 95% confidence interval)	see sections Results: Aim 1 Effects of health literacy and action plan; Results: Aim 2 Effects of allocation method
	17b	For binary outcomes, presentation of both absolute and relative effect sizes is recommended	see section Results: Aim 1: Effects of health literacy and action plan: Secondary outcomes: Perceived difficulty using the action plans;<br>see section Results: Aim 2 Effects of allocation method: Secondary outcomes
Ancillary analyses	18	Results of any other analyses performed, including subgroup analyses and adjusted analyses, distinguishing pre-specified from exploratory	see sections Results: Aim 1 Effects of health literacy and action plan; Results: Aim 2 Effects of allocation method
Harms	19	All important harms or unintended effects in each group (for specific guidance see CONSORT for harms)	NA; stated in abstract as not assessed.
**Discussion**			
Limitations	20	Trial limitations, addressing sources of potential bias, imprecision, and, if relevant, multiplicity of analyses	Discussion paragraph 4
Generalisability	21	Generalisability (external validity, applicability) of the trial findings	Discussion paragraphs 2-3
Interpretation	22	Interpretation consistent with results, balancing benefits and harms, and considering other relevant evidence	Discussion paragraph 1

Other information			
Registration	23	Registration number and name of trial registry	Methods: study design
Protocol	24	Where the full trial protocol can be accessed, if available	Reference 38.
Funding	25	Sources of funding and other support (such as supply of drugs), role of funders	In online submission

*We strongly recommend reading this statement in conjunction with the CONSORT 2010 Explanation and Elaboration for important clarifications on all the items. If relevant, we also recommend reading CONSORT extensions for cluster randomised trials, non-inferiority and equivalence trials, non-pharmacological treatments, herbal interventions, and pragmatic trials. Additional extensions are forthcoming: for those and for up to date references relevant to this checklist, see www.consort-statement.org.

[a]Protocol reference: Ayre J, Cvejic E, Bonner C, Turner RM, Walter SD, McCaffery KJ. Accounting for health literacy and intervention preferences when reducing unhealthy snacking: protocol for an online randomised controlled trial. BMJ Open. 2019;9(5):e028544.

Table C. The TIDieR (Template for Intervention Description and Replication) Checklist*:

Item number	Item	Where located ** Primary paper (page or appendix number)	Other † (details)
	**BRIEF NAME**		
**1.**	Provide the name or a phrase that describes the intervention.	Abstract ('Methods and findings'); Methods ('Allocation method' and 'Action plan intervention' sections	_______________
	**WHY**		
**2.**	Describe any rationale, theory, or goal of the elements essential to the intervention.	Introduction (paragraphs 1-7)	Further text available from [1] (protocol) and [2] (previous study outlining development of literacy-sensitive action plan in greater detail
	**WHAT**		
**3.**	Materials: Describe any physical or informational materials used in the intervention, including those provided to participants or used in intervention delivery or in training of intervention providers. Provide information on where the materials can be accessed (e.g. online appendix, URL).	Appendix (S2)	______________
**4.**	Procedures: Describe each of the procedures, activities, and/or processes used in the intervention, including any enabling or support activities.	Methods (Section Study Design to Measures)	______________
	**WHO PROVIDED**		
**5.**	For each category of intervention provider (e.g. psychologist, nursing assistant), describe their expertise, background and any specific training given.	N/A (delivered through survey platform)	______________
	**HOW**		
**6.**	Describe the modes of delivery (e.g. face-to-face or by some other mechanism, such as internet or telephone) of the intervention and whether it was provided individually or in a group.	Methods ('Study design'; 'Baseline and follow-up surveys')	______________
	**WHERE**		
**7.**	Describe the type(s) of location(s) where the intervention occurred, including any necessary infrastructure or relevant features.	Title; Abstract; Methods (Participants and recruitment)	______________

	**WHEN and HOW MUCH**		
**8.**	Describe the number of times the intervention was delivered and over what period of time including the number of sessions, their schedule, and their duration, intensity or dose.	Method (Baseline and follow-up surveys)	______________
	**TAILORING**		
**9.**	If the intervention was planned to be personalised, titrated or adapted, then describe what, why, when, and how.	Method (Allocation method) – see screened and choice arms; see also description of the action plan interventions (in subsection of allocation methods)	______________
	**MODIFICATIONS**		
**10.‡**	If the intervention was modified during the course of the study, describe the changes (what, why, when, and how).	See Results (Aim 1: Effects of health literacy and action plan) for inclusion of habit strength as covariate	[1] for planned analyses
	**HOW WELL**		
**11.**	Planned: If intervention adherence or fidelity was assessed, describe how and by whom, and if any strategies were used to maintain or improve fidelity, describe them.	Fidelity not assessed as intervention delivered online	______________
**12.‡**	Actual: If intervention adherence or fidelity was assessed, describe the extent to which the intervention was delivered as planned.	Not assessed	______________

** **Authors** - use N/A if an item is not applicable for the intervention being described. **Reviewers** – use '?' if information about the element is not reported/not sufficiently reported.

† If the information is not provided in the primary paper, give details of where this information is available. This may include locations such as a published protocol or other published papers (provide citation details) or a website (provide the URL).

‡ If completing the TIDieR checklist for a protocol, these items are not relevant to the protocol and cannot be described until the study is complete.

* We strongly recommend using this checklist in conjunction with the TIDieR guide (see *BMJ* 2014;348:g1687) which contains an explanation and elaboration for each item.

* The focus of TIDieR is on reporting details of the intervention elements (and where relevant, comparison elements) of a study. Other elements and methodological features of studies are covered by other reporting statements and checklists and have not been duplicated as part of the TIDieR checklist. When a **randomised trial** is being reported, the TIDieR checklist should be used in conjunction with the CONSORT statement (see www.consort-statement.org) as an extension of **Item 5 of the CONSORT 2010 Statement.** When a **clinical trial protocol** is being reported, the TIDieR checklist should be used in conjunction with the SPIRIT statement as an extension of **Item 11 of the SPIRIT 2013 Statement** (see www.spirit-statement.org). For alternate study designs, TIDieR can be used in conjunction with the appropriate checklist for that study design (see www.equator-network.org).

References to other papers related to this study:

[1] Ayre, J., Cvejic, E., Bonner, C., Turner, R. M., Walter, S. D., & McCaffery, K. J. (2019). Accounting for health literacy and intervention preferences when reducing unhealthy snacking: protocol for an online randomised controlled trial. BMJ Open, 9(5), e028544. doi:10.1136/bmjopen-2018-028544

[2] Ayre, J., Bonner, C., Cvejic, E., & McCaffery, K. (2019). Randomized trial of planning tools to reduce unhealthy snacking: Implications for health literacy. PloS one, 14(1), e0209863. doi:10.1371/journal.pone.0209863
