# Supplementary material for: Effects of health literacy, screening, and participant choice on action plans for reducing unhealthy snacking in Australia: A randomised controlled trial
Source: PLoS Med. 2020 Nov 3;17(11):e1003409. doi: 10.1371/journal.pmed.1003409 (PMC7608866; doi:10.1371/journal.pmed.1003409)
Supplement: S2 Text — (PDF) [file pmed.1003409.s004.pdf]

S2 Text: Additional tables

Baseline /descriptive statistics

Tables A-B show descriptive statistics for the full sample (that is, all four allocation method arms, including the arm in which the participant's preference for an action plan was assessed prior to random allocation to an action plan). Totals in Tables C-D reflect only those arms relevant to the accompanying manuscript (that is, the randomized (no preference assessed), choice and screened arms).

Table A: Baseline participant characteristics (categorical variables), all allocation method arms

**Action plan**	**Literacy-sensitive**		**Standard**	
**Demographic variables**	**N**	**%**	**N**	**%**
Female	673	53.3	659	59.5
Speaks English at home	1244	98.6	1095	98.8
Education				
Less than high school education	114	9.0	98	8.8
High school graduate	410	32.5	343	31.0
Certificate	397	31.5	373	33.7
University education	341	27.0	294	26.5
Health literacy (NVS categories)				
Low (scores 0-1)	313	24.8	153	13.8
Moderate (scores 2-3)	388	30.7	190	17.1
High (scores 4-6)	561	44.5	765	69.0
Self-reported BMI (kg/m^2)				
Underweight (<18.5)	5	0.4	1	0.1
Normal weight (18.5-24.9)	46	3.6	37	3.3
Overweight (25.0-29.9)	514	40.7	461	41.6
Obese (≥ 30.0)	697	55.2	609	55.0
Self-reported diagnosis of type 2 diabetes	358	28.4	324	29.2
Self-reported use of insulin	101	28.2	79	24.4
**Total**	**1262**	**53.2**	**1108**	**46.8**

Note. BMI: Body mass index; NVS: Newest Vital Sign

Table B: Baseline participant characteristics (continuous variables), all allocation method arms

Allocation method	Total					
**Action plan**	**Literacy-sensitive**		**Standard**		**Total**	
**Demographic variables**	**M**	**SD**	**M**	**SD**	**M**	**SD**
Age (years)	50.0	11.8	49.3	11.7	**49.7**	**11.7**
Health literacy (NVS; continuous)	3.2	1.9	4.1	1.9	**3.6**	**2.0**
Years since diagnosis (for people who self-reported diabetes)	9.8	8.0	9.3	7.7	**9.6**	**7.9**
Snacking score	25.4	28.7	26.0	29.7	**25.7**	**29.1**
Perceived extent of healthy snacking (1 low – 7 high)	3.8	1.9	3.8	1.9	**3.8**	**1.9**
Perceived extent of unhealthy snacking (1 low – 7 high)	3.4	2.0	3.4	2.0	**3.4**	**2.0**
Habit strength (1 low – 7 high)	4.2	1.4	4.3	1.4	**4.3**	**1.4**
Need for cognition (1 low - 7 high)	4.2	1.0	4.3	1.0	**4.2**	**1.0**
Intention (1 low – 7 high)	5.3	1.3	5.4	1.3	**5.3**	**1.3**

Note. NVS: Newest Vital Sign

Table C: Baseline participant characteristics (continuous variables)

Allocation method	Random						Choice						Screened					
**Action plan**	**Literacy-sensitive**		**Standard**		**Total**		**Literacy-sensitive**		**Standard**		**Total**		**Literacy-sensitive**		**Standard**		**Total**	
**Demographic variables**	**M**	**SD**	**M**	**SD**	**M**	**SD**	**M**	**SD**	**M**	**SD**	**M**	**SD**	**M**	**SD**	**M**	**SD**	**M**	**SD**
Age (years)	49.4	12.2	48.9	11.9	**49.1**	**12.0**	51.0	11.3	48.5	11.9	**50.2**	**11.6**	50.4	11.5	49.6	11.5	**50.0**	**11.5**
Health literacy (NVS; continuous)	3.5	2.0	3.7	2.0	**3.6**	**2.0**	3.7	1.9	3.6	2.0	**3.6**	**1.9**	1.7	1.0	5.2	0.8	**3.6**	**2.0**

Note. NVS: Newest Vital Sign

Table D: Comparison of completer and non-completer baseline characteristics (categorical variables)

Allocation method	Random						Choice						Screened					
Action plan	Completers		Non-Completers		Total		Completers		Non-Completers		Total		Completers		Non-Completers		Total	
**Demographic variables**	**N**	**%**	**N**	**%**	**N**	**%**	**N**	**%**	**N**	**%**	**N**	**%**	**N**	**%**	**N**	**%**	**N**	**%**
Female	213	53.5	121	63.7	**334**	**56.8**	197	52.0	121	59.3	**318**	**54.5**	216	54.0	124	62.6	**340**	**56.9**
Speaks English at home	389	97.7	188	98.9	**577**	**98.1**	375	98.9	201	98.5	**576**	**98.8**	394	98.5	195	98.5	**589**	**98.5**
Education																		
Less than high school education	30	7.5	18	9.5	**48**	**8.2**	37	9.8	18	8.8	**55**	**9.4**	35	8.8	19	9.6	**54**	**9.0**
High school graduate	129	32.4	61	32.1	**190**	**32.3**	111	29.3	69	33.8	**180**	**30.9**	128	32.0	58	29.3	**186**	**31.1**
Certificate	140	35.2	62	32.6	**202**	**34.4**	130	34.3	66	32.4	**196**	**33.6**	131	32.8	61	30.8	**192**	**32.1**
University education	99	24.9	49	25.8	**148**	**25.2**	101	26.6	51	25.0	**152**	**26.1**	106	26.5	60	30.3	**166**	**27.8**
Health literacy (NVS categories)																		
Low (scores 0-1)	79	19.8	31	16.3	**110**	**18.7**	73	19.3	43	21.1	**116**	**19.9**	91	22.8	35	17.7	**126**	**21.1**
Moderate (scores 2-3)	99	24.9	39	20.5	**138**	**23.5**	95	25.1	43	21.1	**138**	**23.7**	97	24.3	44	22.2	**141**	**23.6**
High (scores 4-6)	220	55.3	120	63.2	**340**	**57.8**	211	55.7	118	57.8	**329**	**56.4**	212	53.0	119	60.1	**331**	**55.4**
Self-reported BMI (kg/m^2)																		
Underweight (<18.5)	1	0.3	0	0.0	**1**	**0.2**	1	0.3	3	1.5	**4**	**0.7**	1	0.3	0	0.0	**1**	**0.2**
Normal weight (18.5-24.9)	12	3.0	10	5.3	**22**	**3.7**	7	1.8	8	3.9	**15**	**2.6**	19	4.8	6	3.0	**25**	**4.2**
Overweight (25.0-29.9)	168	42.2	68	35.8	**236**	**40.1**	155	40.9	90	44.1	**245**	**42.0**	161	40.3	75	37.9	**236**	**39.5**
Obese (≥ 30.0)	217	54.5	112	58.9	**329**	**56.0**	216	57.0	103	50.5	**319**	**54.7**	219	54.8	117	59.1	**336**	**56.2**
Self-reported diagnosis of type 2 diabetes	104	26.1	65	34.2	**169**	**28.7**	85	22.4	87	42.6	**172**	**29.5**	104	26.0	78	39.4	**182**	**30.4**
Self-reported use of insulin	28	26.9	15	23.1	**43**	**25.4**	21	24.7	20	23.0	**41**	**23.8**	26	25.0	25	32.1	**51**	**28.0**
**Total**	**398**	**67.7**	**190**	**32.3**	**588**		**379**	**65.0**	**204**	**35.0**	**583**		**400**	**66.9**	**198**	**33.1**	**598**	

Note. BMI: Body mass index; NVS: Newest Vital Sign

Table E: Comparison of completer and non-completer baseline characteristics (continuous variables)

Allocation method	Random						Choice						Screened					
**Action plan**	**Completers**		**Non-Completers**		**Total**		**Completers**		**Non-Completers**		**Total**		**Completers**		**Non-Completers**		**Total**	
**Demographic variables**	**M**	**SD**	**M**	**SD**	**M**	**SD**	**M**	**SD**	**M**	**SD**	**M**	**SD**	**M**	**SD**	**M**	**SD**	**M**	**SD**
Age (years)	49.7	11.7	47.9	12.7	**49.1**	**12.0**	50.3	11.4	49.8	11.9	**50.2**	**11.6**	50.5	10.9	48.9	12.4	**50.0**	**11.5**
Health literacy (NVS; continuous)	3.6	2.0	3.8	1.9	**3.6**	**2.0**	3.6	1.9	3.7	1.9	**3.6**	**1.9**	3.5	2.0	3.8	2.0	**3.6**	**2.0**
Years since diagnosis (for people who self-reported diabetes)	9.5	7.3	9.5	7.9	**9.5**	**7.5**	8.9	7.9	8.7	7.8	**8.8**	**7.8**	10.4	8.4	10.4	7.2	**10.4**	**7.9**
Snacking score	26.2	39.5	29.8	34.1	**27.4**	**37.9**	24.5	30.1	25.7	29.2	**24.9**	**29.7**	23.7	27.4	28.5	44.8	**25.3**	**34.2**
Perceived extent of healthy snacking (1 low – 7 high)	3.7	2.0	3.7	1.9	**3.7**	**2.0**	3.7	1.9	3.7	1.8	**3.7**	**1.9**	4.0	2.0	4.1	1.9	**4.0**	**2.0**
Perceived extent of unhealthy snacking (1 low – 7 high)	3.4	2.0	3.8	2.0	**3.5**	**2.0**	3.4	2.0	3.4	2.0	**3.4**	**2.0**	3.2	1.9	3.1	2.0	**3.2**	**2.0**
Habit strength (1 low – 7 high)	4.3	1.5	4.2	1.3	**4.3**	**1.4**	4.2	1.4	4.3	1.4	**4.2**	**1.4**	4.3	1.4	4.3	1.5	**4.3**	**1.4**
Need for cognition (1 low - 7 high)	4.2	0.9	4.2	1.1	**4.2**	**1.0**	4.2	1.0	4.3	1.1	**4.2**	**1.1**	4.2	1.0	4.2	1.0	**4.2**	**1.0**
Intention (1 low – 7 high)	5.3	1.4	5.5	1.2	**5.4**	**1.3**	5.2	1.3	5.5	1.3	**5.3**	**1.3**	5.3	1.3	5.5	1.3	**5.4**	**1.3**

Note. NVS: Newest Vital Sign

Research question 1: Action plan and health literacy interaction

Primary outcome

All observations

Per protocol analysis

Table F: Multiple linear regression model predicting reduction in serves of unhealthy snacks per week (randomized arm, all observations)[a]

Predictor	Unadjusted		Adjusted[b]	
	**B (95% CI)**	**P value**	**B (95% CI)**	**P value**
(Intercept)	1.54 (-3.54, 6.61)	0.553	-0.02 (-4.90, 4.86)	0.994
Baseline snacking score	0.47 (0.38, 0.55)	<0.001	0.54 (0.44, 0.63)	<0.001
Health literacy (NVS)	3.88 (1.30, 6.47)	0.003	4.21 (1.73, 6.69)	0.001
Action plan = literacy-sensitive action plan[c]	0.77 (-6.12, 7.65)	0.827	3.53 (-3.12, 10.18)	0.299
Action plan = literacy-sensitive action plan × health literacy (NVS)	-3.20 (-6.66, 0.25)	0.069	-3.25 (-6.55, 0.05)	0.054
Age (years)	–		0.39 (0.08, 0.69)	0.012
English spoken at home	–		20.11 (2.15, 42.37)	0.077
Education	–		-3.35 (-7.01, 0.32)	0.073
Self-reported diabetes status	–		-10.03 (-17.79, -2.27)	0.011
Baseline habit strength	–		5.10 (2.61, 7.59)	<0.001

Note. NVS: Newest Vital Sign; [a]analysis uses only participants allocated to the arm: 'random (prior preference not assessed)'; [b]Adjusted analysis controls for mean-centred baseline snacking score, age, English spoken at home, education, self-reported diabetes status, and baseline habit strength; [c]action plans were coded such that 1=literacy-sensitive, 0=standard.

Intention-to-treat analysis

For intention-to-treat analyses, reduction in snacking scores at one-month follow-up was estimated for best- and worst- case scenarios:

1. Best-case scenario: difference score = mean difference score for that combination of intervention group and allocation method
2. Worst-case scenario: difference score = 0 (i.e. no change from baseline).

Best-case scenario

Table G: Multiple linear regression model predicting reduction in serves of unhealthy snacks per week (best-case scenario, randomized arm, all observations)[a]

Predictor	Unadjusted		Adjusted[b]	
	B (95% CI)	P value	B (95% CI)	P value
(Intercept)	2.35 (-2.30, 7.00)	0.323	-0.57 (-3.93, 5.07)	0.804
Baseline snacking score	0.42 (0.34, 0.49)	<0.001	0.48 (0.40, 0.56)	<0.001
Health literacy (NVS)	3.72 (1.34, 6.10)	0.002	4.00 (1.70, 6.29)	0.001
Action plan = literacy-sensitive action plan[c]	0.20 (-5.69, 6.08)	0.948	2.95 (-2.78, 8.67)	0.313
Action plan = literacy-sensitive action plan × health literacy (NVS)	-3.72 (-1.34, 6.10)	0.048	-3.11 (-5.99, 0.23)	0.034
Age (years)	–		0.32 (0.07, 0.57)	0.011
English spoken at home	–		21.12 (1.62, 40.61)	0.034
Education	–		-2.56 (-5.61, 0.48)	0.099
Self-reported diabetes status	–		-8.44 (-14.80, -2.07)	0.009
Baseline habit strength	–		4.64 (2.49, 6.79)	<0.001

Note. NVS: Newest Vital Sign; [a]analysis uses only participants allocated to the arm: 'random (prior preference not assessed)'; [b]Adjusted analysis controls for mean-centred baseline snacking score, age, English spoken at home, education, self-reported diabetes status, and baseline habit strength; [c]action plans were coded such that 1=literacy-sensitive, 0=standard.

Worst-case scenario

Table H: Multiple linear regression model predicting reduction in serves of unhealthy snacks per week (worst-case scenario, randomized arm, all observations)[a]

Predictor	Unadjusted		Adjusted[b]	
	B (95% CI)	P value	B (95% CI)	P value
(Intercept)	1.13 (-2.31, 4.57)	0.520	-0.23 (-3.10, 3.56)	0.894
Baseline snacking score	0.34 (0.28,0.40)	<0.001	0.41 (0.34, 0.47)	<0.001
Health literacy (NVS)	2.30 (0.55, 4.05)	0.010	2.53 (0.84, 4.22)	0.003
Action plan = literacy-sensitive action plan[c]	0.69 (-4.12, 5.49)	0.780	2.50 (-2.18, 7.18)	0.295
Action plan = literacy-sensitive action plan × health literacy (NVS)	-1.81 (-4.24, 0.63)	0.147	-1.81 (-4.16, 0.54)	0.132
Age (years)	–		0.28 (0.07, 0.49)	0.010
English spoken at home	–		21.72 (4.49, 38.95)	0.013
Education	–		-2.55 (-5.11, 0.01)	0.051
Self-reported diabetes status	–		-8.67 (-14.01, -3.32)	0.001
Baseline habit strength	–		4.12 (2.29, 5.94)	<0.001

Note. NVS: Newest Vital Sign; [a]analysis uses only participants allocated to the arm: 'random (prior preference not assessed)'; [b]Adjusted analysis controls for mean-centred baseline snacking score, age, English spoken at home, education,

self-reported diabetes status, and baseline habit strength; [c]action plans were coded such that 1=literacy-sensitive, 0=standard.

Outlier and influential observations removed

Per protocol analysis

Table I: Multiple linear regression model predicting reduction in serves of unhealthy snacks per week (randomized arm, outlier and influential observations removed)[a]

Predictor	Unadjusted		Adjusted[b]	
	B (95% CI)	P value	B (95% CI)	P value
(Intercept)	3.48 (0.78, 6.17)	0.011	3.02 (0.39, 5.65)	0.024
Baseline snacking score	0.63 (0.58, 0.68)	<0.001	0.69 (0.63, 0.74)	<0.001
Health literacy (NVS)	1.98 (0.60, 3.37)	0.005	1.99 (0.64, 3.34)	0.004
Action plan = literacy-sensitive action plan[c]	1.57 (-2.11, 5.25)	0.403	2.44 (-1.17, 6.04)	0.186
Action plan = literacy-sensitive action plan × health literacy (NVS)	-2.11 (-3.09, -0.24)	0.027	-2.07 (-3.89, -0.26)	0.025
Age (years)	–		0.09 (0.07, 0.26)	0.274
English spoken at home	–		3.31 (-9.27, 15.88)	0.606
Education	–		1.20 (-0.80, 3.19)	0.240
Self-reported diabetes status	–		-0.82 (-5.07, 3.43)	0.706
Baseline habit strength	–		3.21 (1.86, 4.57)	<0.001

Note. NVS: Newest Vital Sign; [a]analysis uses only participants allocated to the arm: 'random (prior preference not assessed)'; [b]Adjusted analysis controls for mean-centred baseline snacking score, age, English spoken at home, education, self-reported diabetes status, and baseline habit strength; [c]action plans were coded such that 1=literacy-sensitive, 0=standard.

Action plan characteristics

Table J: Frequency of situations and solutions selected by participants using the literacy-sensitive action plan in the randomized arm

Scenario selected for 'if statement'	N	%	Solution selected for 'then statement'	N	%
I have a craving	56	18.5	eat a piece of fruit	87	28.8
I am bored	46	15.2	drink a large glass of water	75	24.8
I start with one piece but then keep eating	36	11.9	eat fresh vegetables and dip	25	8.3
I am in front of a TV or computer	35	11.6	eat a smaller amount	23	7.6
I am busy or stressed	21	7.0	do a chore or task	22	7.3
I am sad	17	5.6	drink tea	16	5.3
Other scenarios with frequency <5% *	91	30.1	go for a walk	16	5.3
			Other solutions with frequency <5%†	38	12.6

Note.
*'Other scenarios with frequency <5%': 'people around me are eating', 'the snack is right in front of me', 'I am drinking tea or coffee', 'I am drinking alcohol', 'it is part of a celebration or special event', 'I have arrived home', 'someone offers me a snack', 'I want to reward myself' , 'I am happy.'
†'Other solutions with frequency <5%' include: 'listen to music,' 'chat to someone for 5 minutes', 'take the food out of the packet and put it on a plate,' 'move the snack into the cupboard,' 'politely say "No thank you",' and 'take a break.'

Table K: Characteristics of standard action plans by health literacy level, randomized arm

Plan characteristic	Low/moderate health literacy		High health literacy		Total	
	N	%	N	%	N	%
**Extent that plan reflects instructions**						
No plan created*	32	28.1	5	2.9	37	12.9
Plan was not specific (e.g. "eat less junk food")*	16	14.0	13	7.6	29	10.1
Plan identified a situation for unhealthy snacking	48	42.1	131	76.2	179	62.6
Plan identified a solution for unhealthy snacking	66	57.9	155	90.1	221	77.3
**Additional characteristics**						
Plan identified situation equivalent to an option available in literacy-sensitive action plan	19	16.7	58	33.7	77	26.9
Plan identified situation not listed in literacy-sensitive action plan	30	26.3	98	57.0	128	44.8
Plan identified a personal solution equivalent to an option available in literacy-sensitive action plan	43	37.7	112	65.1	155	54.2
Plan identified a personal solution not presented in literacy-sensitive action plan	51	44.7	113	65.7	164	57.3
Content of described solutions						
Eating a healthy snack	36	31.6	98	57.0	134	46.9
Preparing healthy snacks ahead of time	18	15.8	45	26.2	63	22.0
Removing tempting foods (incl not buying them)	16	14.0	33	19.2	49	17.1

Scheduling meals and snack, and meal plans	7	6.1	20	11.6	27	9.4
Drink a glass of water	8	7.0	19	11.0	27	9.4
Restricting specific tempting unhealthy snacks	8	7.0	17	9.9	25	8.7
Distraction	4	3.5	14	8.1	18	6.3
Exercise	6	5.3	7	4.1	13	4.5
Self-talk (e.g. "remind myself"/"think positive")	2	1.8	7	4.1	9	3.1
Other	18	15.8	32	18.6	50	17.5
**Total number of participants**	**114**		**172**		**286**	

Note. *These categories were mutually exclusive.

Research question 2: Allocation method

Primary outcome

All observations

Per protocol analysis

Table L: Multiple linear regression model predicting reduction in serves of unhealthy snacks per week, by allocation arm, all observations

	**All observations**			
**Predictor**	**Unadjusted**		**Adjusted[a]**	
	**B (95% CI)**	**P value**	**B (95% CI)**	**P value**
(Intercept)	2.14 (0.54, 3.74)	0.009	1.39 (-1.06, 3.84)	0.267
Baseline snacking score	0.37 (0.32, 0.41)	<0.001	0.45 (0.39, 0.50)	<0.001
Health literacy (NVS)	1.28 (0.47, 2.10)	0.002	1.47 (0.61, 2.32)	0.001
Allocation method contrast 1: Randomised vs Choice/Screened)	0.15 (-2.11, 2.40)	0.908	0.16 (-2.04, 2.37)	0.898
Allocation method contrast 2: Choice vs screened	1.89 (-0.07, 3.86)	0.057	1.79 (-0.16, 3.73)	0.067
Action plan = literacy-sensitive action plan[b]	–		0.79 (-2.61, 4.19)	0.633
Age (years)	–		0.33 (0.19, 0.48)	<0.001
English spoken at home	–		5.66 (-6.78, 18.10)	0.374
Education	–		-0.81 (-2.52, 0.89)	0.343
Self-reported diabetes status	–		-6.07 (-9.79, -2.35)	0.001
Baseline habit strength	–		3.38 (2.17, 4.60)	<0.001

Note. NVS: Newest Vital Sign; [a]Adjusted analysis controls for mean-centred baseline snacking score, age, English spoken at home, education, self-reported diabetes status, and baseline habit strength; [b]action plans were coded such that 1=literacy-sensitive, 0=standard.

Intention-to-treat analysis: Best-case scenario

Table M: Multiple linear regression model predicting reduction in serves of unhealthy snacks per week (best-case scenario, all observations)

Predictor	Unadjusted		Adjusted[a]	
	B (95% CI)	P value	B (95% CI)	P value
(Intercept)	2.04 (0.53, 3.55)	0.008	1.46 (-0.91, 3.83)	0.226
Baseline snacking score	0.35 (0.30, 0.39)	<0.001	0.42 (0.37, 0.47)	<0.001
Health literacy (NVS)	1.23 (0.46, 2.00)	0.002	1.38 (0.58, 2.18)	0.001
Allocation method contrast 1: Randomised vs Choice/Screened)	0.02 (-2.03, 2.08)	0.982	0.16 (-1.86, 2.18)	0.875
Allocation method contrast 2: Choice vs screened	1.88 (-0.02, 3.79)	0.053	1.77 (-0.12, 3.66)	0.067
Action plan = literacy-sensitive action plan[b]	–		0.66 (-2.53, 3.84)	0.686
Age (years)	–		0.31 (0.17, 0.45)	<0.001
English spoken at home	–		6.21 (-5.56, 17.99)	0.301
Education	–		-0.67 (-2.26, 0.93)	0.414
Self-reported diabetes status	–		-5.58 (-9.04, -2.11)	0.002
Baseline habit strength	–		3.22 (2.07, 4.37)	<0.001

Note. NVS: Newest Vital Sign; [a]Adjusted analysis controls for mean-centred baseline snacking score, age, English spoken at home, education, self-reported diabetes status, and baseline habit strength; [b]action plans were coded such that 1=literacy-sensitive, 0=standard.

Intention-to-treat analysis: Worst-case scenario

Table N: Multiple linear regression model predicting reduction in serves of unhealthy snacks per week (worst-case scenario, all observations)

Predictor	Unadjusted		Adjusted[a]	
	B (95% CI)	P value	B (95% CI)	P value
(Intercept)	1.13 (0.03, 2.23)	0.045	0.96 (-0.70, 2.63)	0.257
Baseline snacking score	0.22 (0.19, 0.26)	<0.001	0.28 (0.24, 0.31)	<0.001
Health literacy (NVS)	0.81 (0.24, 1.38)	0.005	0.93 (0.34, 1.53)	0.002
Allocation method contrast 1: Randomised vs Choice/Screened)	0.09 (-1.47, 1.66)	0.906	0.10 (-1.44, 1.63)	0.903
Allocation method contrast 2: Choice vs screened	1.10 (-0.25, 2.45)	0.111	1.04 (-0.31, 2.39)	0.130
Action plan = literacy-sensitive action plan[b]	–		0.38 (-1.96, 2.73)	0.749
Age (years)	–		0.23 (0.13, 0.33)	<0.001
English spoken at home	–		7.78 (-1.10, 16.65)	0.086
Education	–		-0.93 (-2.10, 0.24)	0.120
Self-reported diabetes status	–		-4.82 (-7.28, -2.36)	<0.001
Baseline habit strength	–		2.22 (1.37, 3.07)	<0.001

Note. NVS: Newest Vital Sign; [a]Adjusted analysis controls for mean-centred baseline snacking score, age, English spoken at home, education, self-reported diabetes status, and baseline habit strength; [b]action plans were coded such that 1=literacy-sensitive, 0=standard.

Outlier and influential observations removed

Per protocol analysis

Table O: Multiple linear regression model predicting reduction in serves of unhealthy snacks per week (per protocol analysis, outlier and influential observations removed)

Predictor	Unadjusted		Adjusted[a]	
	B (95% CI)	P value	B (95% CI)	P value
(Intercept)	4.39 (3.44, 5.34)	<0.001	4.62 (3.17, 6.07)	<0.001
Baseline snacking score	0.55 (0.51, 0.58)	<0.001	0.62 (0.58, 0.66)	<0.001
Health literacy (NVS)	0.15 (-0.34, 0.63)	0.592	0.16 (-0.35, 0.67)	0.553
Allocation method contrast 1: Randomised vs Choice/Screened)	0.62 (-0.72, 1.95)	0.374	0.55 (-0.76, 1.85)	0.421
Allocation method contrast 2: Choice vs screened	0.69 (-0.47, 1.85)	0.235	0.45 (-0.71, 1.59)	0.432
Action plan = literacy-sensitive action plan[b]	–		-0.41 (-2.43, 1.60)	0.719
Age (years)	–		0.13 (0.04, 0.22)	0.004
English spoken at home	–		3.81 (-3.68, 11.30)	0.321
Education	–		0.83 (-0.18, 1.84)	0.117
Self-reported diabetes status	–		-0.32 (-2.54, 1.90)	0.763
Baseline habit strength	–		2.58 (1.84, 3.33)	0.001

Note. NVS: Newest Vital Sign; [a]Adjusted analysis controls for mean-centred baseline snacking score, age, English spoken at home, education, self-reported diabetes status, and baseline habit strength; [b]action plans were coded such that 1=literacy-sensitive, 0=standard.

Secondary outcomes

Research question 1: All observations, per protocol analysis

Table P: Multiple linear regression model predicting perceived extent of unhealthy snacking at follow-up (randomized arm, all observations)[a]

Predictor	Unadjusted		Adjusted[b]	
	B (95% CI)	P value	B (95% CI)	P value
(Intercept)	3.39 (3.15, 3.62)	<0.001	3.39 (3.16, 3.61)	<0.001
Baseline perceived extent of unhealthy snacking[c]	0.60 (0.52, 0.67)	<0.001		
Health literacy (NVS)	-0.05 (-0.17, 0.07)	0.402	-0.03 (-0.15, 0.08)	0.550
Action plan = literacy-sensitive action plan[c]	0.07 (-0.25, 0.39)	0.663	0.02 (-0.29, 0.33)	0.889
Action plan = literacy-sensitive action plan × health literacy (NVS)	0.01 (-0.15, 0.17)	0.896	0.01 (-0.14, 0.17)	0.870
Age (years)			-0.02 (-0.04, -0.01)	0.001
English spoken at home			-0.15 (-1.18, 0.87)	0.768
Education			-0.10 (-0.27, 0.07)	0.256
Self-reported diabetes status			0.17 (-0.19, 0.54)	0.349
Baseline habit strength			-0.37 (-0.52, -0.21)	<0.001

Note. NVS: Newest Vital Sign; [a]analysis uses only participants allocated to the arm: 'random (prior preference not assessed)'; [b]Adjusted analysis controls for mean-centred baseline perceived extent of unhealthy snacking, age, English spoken at home, education, self-reported diabetes status, and baseline habit strength; [c]action plans were coded such that 1=literacy-sensitive, 0=standard.

Research question 2: All observations, per protocol analysis

Tables S17-S19 show analysis of secondary outcomes for Research Question 2 (per protocol, all observations). There was no main effect of allocation method on any secondary outcomes (perceived extent of unhealthy snacking at follow-up: ($\chi^2(2)$ =1.22, p=0.54); habit strength ($\chi^2(2)$ =0.99, p=0.61); action control ($\chi^2(2)$ =3.67, p=0.16). Coefficients for the allocation method contrasts and other variables are shown in the tables below.

Table Q: Multiple linear regression model predicting perceived extent of unhealthy snacking at follow-up (per protocol analysis, all observations)

Predictor	Unadjusted		Adjusted[a]	
	B (95% CI)	P value	B (95% CI)	P value
(Intercept)	3.32 (3.22, 3.41)	<0.001	3.31 (3.17, 3.44)	<0.001
Baseline perceived extent of unhealthy snacking	0.6 (0.55, 0.64)	<0.001	0.38 (0.32, 0.45)	<0.001
Health literacy (NVS)	-0.03 (-0.07, 0.02)	0.235	-0.01 (-0.06, 0.04)	0.629

Allocation method contrast 1: Randomised vs Choice/Screened)	0.03 (-0.10, 0.16)	0.620	0.04 (-0.09, 0.16)	0.558
Allocation method contrast 2: Choice vs screened	0.04 (-0.07, 0.15)	0.479	0.05 (-0.06, 0.16)	0.345
Action plan = literacy-sensitive action plan[b]			0.02 (-0.17, 0.21)	0.810
Age (years)			-0.02 (-0.03, -0.01)	<0.001
English spoken at home			-0.37 (-1.07, 0.33)	0.300
Education			-0.05 (-0.14, 0.05)	0.352
Self-reported diabetes status			0.01 (-0.20, 0.22)	0.948
Baseline habit strength			-0.36 (-0.45, -0.28)	<0.001

Note. NVS: Newest Vital Sign; [a]Adjusted analysis controls for mean-centred baseline perceived extent of unhealthy snacking, age, English spoken at home, education, self-reported diabetes status, and baseline habit strength; [b]action plans were coded such that 1=literacy-sensitive, 0=standard.

Table R: Multiple linear regression model predicting habit strength at follow-up (per protocol analysis, all observations)

Predictor	Unadjusted		Adjusted[a]	
	B (95% CI)	P value	B (95% CI)	P value
(Intercept)	4.26 (4.2, 4.31)	<0.001	4.24 (4.16, 4.33)	<0.001
Health literacy (NVS)	0.04 (0.01, 0.07)	0.005	0.04 (0.01, 0.07)	0.011
Allocation method contrast 1: Randomised vs Choice/Screened)	-0.01 (-0.09, 0.07)	0.810	0.00 (-0.08, 0.07)	0.920
Allocation method contrast 2: Choice vs screened	-0.03 (-0.10, 0.03)	0.307	-0.03 (-0.10, 0.03)	0.326
Action plan = literacy-sensitive action plan[b]			0.01 (-0.10, 0.13)	0.841
Age (years)			0.02 (0.01, 0.02)	0.057
English spoken at home			0.42 (-0.01, 0.85)	0.057
Education			0.05 (-0.01, 0.11)	0.094
Self-reported diabetes status			-0.03 (-0.15, 0.10)	0.692
Baseline habit strength	0.72 (0.68, 0.76)	<0.001	0.69 (0.65, 0.73)	<0.001

Note. NVS: Newest Vital Sign; [a]Adjusted analysis controls for mean-centred baseline habit strength, age, English spoken at home, education, and self-reported diabetes status; [b]action plans were coded such that 1=literacy-sensitive, 0=standard.

Table S: Multiple linear regression model predicting action control (per protocol analysis, all observations)

Predictor	Unadjusted		Adjusted[a]	
	B (95% CI)	P value	B (95% CI)	P value
(Intercept)	4.58 (4.50, 4.65)	<0.001	4.63 (4.52, 4.75)	<0.001
Health literacy (NVS)	0.05 (0.01, 0.09)	0.008	0.04 (0, 0.08)	0.065
Allocation method contrast 1: Randomised vs Choice/Screened)	-0.08 (-0.18, 0.03)	0.173	-0.08 (-0.19, 0.02)	0.126
Allocation method contrast 2: Choice vs screened	-0.03 (-0.12, 0.07)	0.565	-0.06 (-0.15, 0.04)	0.243
Action plan = literacy-sensitive action plan[b]			-0.1 (-0.26, 0.07)	0.251
Age (years)			0.01 (0, 0.02)	0.015
English spoken at home			0.45 (-0.15, 1.04)	0.139
Education			0.1 (0.02, 0.18)	0.015
Self-reported diabetes status			0.14 (-0.04, 0.32)	0.118
Baseline habit strength			0.18 (0.13, 0.23)	<0.001

Note. NVS: Newest Vital Sign; [a]Adjusted analysis controls for mean-centred age, English spoken at home, education, self-reported diabetes status, and baseline habit strength; [b]action plans were coded such that 1=literacy-sensitive, 0=standard.
