# Supplementary material for: Effects of health literacy, screening, and participant choice on action plans for reducing unhealthy snacking in Australia: A randomised controlled trial
Source: PLoS Med. 2020 Nov 3;17(11):e1003409. doi: 10.1371/journal.pmed.1003409 (PMC7608866; doi:10.1371/journal.pmed.1003409)
Supplement: Attachment — Submitted filename: ayre.pdf [file pmed.1003409.s005.pdf]

This paper reports a three arm trial of interventions to reduce unhealthy snacking in Australia.

I was asked for a statistical report and I interpret that to include all aspects of the design and conduct of the study.

# Points of detail

**Page 14** I found this section unclear. Later we learn that the maximum snacking score was 630 per week. If my calculations are correct this must mean they were asked about nine different types of snack since $9 * 10 * 7 = 630$ but this is not stated here. If I am incorrect about what was asked then even more reason to clarify.

**Page 15** The disadvantage of the method of imputation chosen here is that both the worst and best case impute the same score for a large number of people which affects the variability. Having said that it is hard to see multiple imputation working well here and the authors have gone the extra kilometre here as most people use what they call modified intention to treat: an analysis including all those with at least one post randomisation observation..

**Page 18** Confidence intervals for differences might be a useful addition here and perhaps odds ratios with confidence intervals for the categorical predictors of dropout. I do not understand why the denominator degrees of freedom for one of the F is so different from the others.

**Page 18** I think all the predictors of missingness should be in the model and I do not think they currently are.

**Page 24** While I agree with the main thrust of the first paragraph here I would question the use of the word still in the phrase ‘there was still evidence’.

**Page 26** The sentence starting ‘there was no overall effect’ omits what it was effect on.

# Points of more substance

## Influential points

The issue of what to do with influential points is always a complex one unless there is a clear explanation of why they have occurred.

- Presumably it is the changes which are relevant her, not the baseline or follow–up values as that is what is being modelled?
- Are these values as plausible as the authors suggest? I am not that familiar with nutrition data but it looks as though they are comparable with the calorific intake I remember the track cyclist Chris Hoy reporting when he was training for the Olympics (and the current figures do not include non–snacks).
- Rather than excluding them what happens is the authors either treat them as missing and then impute them or if they winsorise the top X% `https://en.wikipedia.org/wiki/Winsorizing`.
- Excluding different numbers of people (eight on page 24 and twenty–four on page 27) is very data–driven and it would be better to identify a common group of suspicious observations across the analysis.

The problem is that the models are clearly heavily influenced by the subset of observations so those observations do need detailed attention.

## Structure

I found the overall structure of the paper confusing. This is because the two aims are presented together. If I understand this correctly aim 1 is about trying to replicate whether the model holds in a new sample. Aim 2 is the comparison of what the trial was set up to do: compare the arms. The second seems more important to me as it extends the theory in a novel way but is in danger of being lost. At the very least I think we need clearer signposting as to what the two models are showing us. I would also consider putting aim 2 first and then introducing aim 1 as a test of fidelity. Another option would be to remove aim 1 to supplementary material but this might be a bit strong. Of course if I have not understood what aims 1 and 2 are doing then even more reason to try to clarify as other readers may be equally dim.

### The unreported arm

In principle I think all the arms should be reported in one paper but that is more of an editorial decision. Given the confusion I have already confessed to about the two aims in the present version having a third one may be the straw which breaks the camel's back.

## Summary

Despite the length of this review I do not have major concerns. Obviously the issue of what snacks those missing at follow–up were consuming is important but unknowable.

Michael Dewey
