# Supplementary material for: Effects of health literacy, screening, and participant choice on action plans for reducing unhealthy snacking in Australia: A randomised controlled trial
Source: PLoS Med. 2020 Nov 3;17(11):e1003409. doi: 10.1371/journal.pmed.1003409 (PMC7608866; doi:10.1371/journal.pmed.1003409)
Supplement: Attachment — Submitted filename: PMEDICINE-D-20-00842R1_Manuscript Review.pdf [file pmed.1003409.s006.pdf]

Manuscript ID: PMEDICINE-D-20-00842R1

Manuscript Title: Effects of health literacy, screening and participant choice on action plans for reducing unhealthy snacking: A randomised controlled trial

Category	Reviewer Comments
**Consistency with Journal Mission**	This study aligns with the mission of this journal because of the paper's potential to inform clinical practice related to tailoring action planning to the health literacy of an individual. This paper also has the future potential to help clinicians and intervention sciences improve risk factors to enhance the overall quality of life by tailoring intervention action plans to a person's health literacy level.
**Background Literature Review**	I would suggest adding more background literature on the "universal precautions" approach. This would provide the reader with more insight into what this approach is and how it has been used in health interventions.
**Theoretical Basis**	**N/A**
**Methods/Strategies/Intervention Applications**	I would suggest explaining why participants with diabetes were recruited first followed by expanding the participant pool to people with who were considered overweight or obese. In Figure 1 for the allocation method, I would encourage the authors to make sure the names of the allocation methods coincided with the allocation methods in the paper narrative. For example, in the paper, the allocation method is "Random (Arm A2)" but in the figure, the allocation method for is "Random (Arm A)". I would suggest adding the "2" so they would be the same.<br>Please include the length of the intervention and how often did the participants have to engage in the intervention. Also, was race considered? If so, I would suggest including this information in your paper. When intervention materials were designed was health literacy considered? If so, please add why or why not. This could provide more insight on your findings.
**Discussion/Conclusions**	Overall, very clear and connected to back findings. I would suggest discussing how the practical application of your results could aid in future intervention testing and clinical application.
**Additional Comments**	The strengths of this article were the detailed approach to significance of health literacy and its impact on action planning with diabetic and overweight/obese individuals. The tables and figures were also well-detailed and connected to the research strategy. The weaknesses of this paper are that the authors did not address the ethnic/racial factors or barriers that individuals with low health literacy typically face when engaging in health related initiatives. Leaving the external generalizability of this study limiting. Overall, the authors did a great job of connecting each aim and discussing the importance of why health literacy should be taken into consideration. I would suggest minor revisions to enhance the quality of the paper.
