# Supplementary material for: Effects of health literacy, screening, and participant choice on action plans for reducing unhealthy snacking in Australia: A randomised controlled trial
Source: PLoS Med. 2020 Nov 3;17(11):e1003409. doi: 10.1371/journal.pmed.1003409 (PMC7608866; doi:10.1371/journal.pmed.1003409)
Supplement: Attachment — Submitted filename: response to reviewers.pdf [file pmed.1003409.s007.pdf]

THE UNIVERSITY OF SYDNEY

Ms Julie Ayre
Sydney School of Public Health, The University of Sydney
Monday, June 1st, 2020

Dear Dr Veitch,
RE: PLOS Medicine: Effects of health literacy, screening and participant choice on action plans for reducing unhealthy snacking: A randomised controlled trial" (PMEDICINE-D-20-00842R1)

Thank you very much for the opportunity to revise our article submitted to PLOS Medicine. We have read the comments of the reviewers and editors with interest, and have revised our paper in light of these comments. We have addressed each comment, with full details of the revisions given below in bold. These are also shown in tracked changes in the manuscript document. We have also checked to make sure images adhere to requirements and were uploaded to PACE. An open-access protocol has already been published (https://bmjopen.bmj.com/content/9/5/e028544) and is referred to in-text.

We trust that our adjustments make the paper suitable for publication in PLOS Medicine, and we look forward to your final decision.

Kind regards,

Julie Ayre,
On behalf of all authors

Requests from the editors:

1. *In the last sentence of the Abstract Methods and Findings section, please include a brief description of any key limitation(s) of the study/study methods.

Response: We have added that *'Key limitations include low-moderate retention and use of a single-occasion self-reported primary outcome'* to the Methods and Findings section of the abstract (p2).

Comments from the reviewers:
Reviewer #1:
Points of detail:

1. Page 14 I found this section unclear. Later we learn that the maximum snacking score was 630 per week. If my calculations are correct this must mean they were asked about nine different types of snack since $9 * 10 * 7 = 630$ but this is not stated here. If I am incorrect about what was asked then even more reason to clarify.

   Response: We have amended the section detailing measurement of snacking to explain that there were 9 categories of discretionary foods. You were correct in understanding that maximum number of snacks possible using the instrument was 9 categories of snacks * 10 maximum serves * 7 days per week (p13):

   *"Participants were asked to reflect on their snacking behavior in the past month **for nine categories of discretionary foods**. Participants selected a timeframe (day, week, month or 'I don't eat this') and indicated the number of serves for this timeframe (up to a maximum ten*

serves).”

2. Page 15 The disadvantage of the method of imputation chosen here is that both the worst and best case impute the same score for a large number of people which affects the variability. Having said that it is hard to see multiple imputation working well here and the authors have gone the extra kilometre here as most people use what they call modified intention to treat: an analysis including all those with at least one post randomisation observation.

   Response: Thank you for acknowledging this rigour in our study.

3. Page 18 Confidence intervals for differences might be a useful addition here and perhaps odds ratios with confidence intervals for the categorical predictors of dropout. I do not understand why the denominator degrees of freedom for one of the F is so different from the others.

   Response: The difference in the degrees freedom mentioned above relates only to the randomised arm, whereas the other degrees of freedom in that passage refer to all three allocation method arms collectively. On reflection of the reviewer’s 4th comment (see below), the latter is less important than describing differences in completion rates for other variables that were tested (across the three arms), even if there was no evidence of a difference. This change is reflected as below. Odds ratios and confidence intervals have now also been added as requested (p17):

   “Overall, 66.5% (N=1,177) of the participants completed follow-up. There was no difference in completion rates across arms ($\chi^2(2)$ =0.99, p=0.61) (S1 Table S4). Participants lost to follow-up were more likely to be female (53.2% of non-completers compared to 61.8% of completers; $\chi^2(1)$ =11.93, p<0.001; ***OR 1.43 (95% CI: 1.17 to 1.74)****), and were more likely to report a diagnosis of type 2 diabetes (38.9% of non-completers compared to 24.9% of completers; $\chi^2(1)$ =36.85, p=0.001;* ***OR 1.92 (95% CI: 1.55 to 2.37)****) (S1 Table S4). On average, participants lost to follow-up were also younger (-1.3 years; $F_{(1,1767)}$=5.1, p=0.03;* ***95% CI: -2.48 to -0.17****), and reported stronger intentions to reduce their unhealthy snacking (+0.2; $F_{(1,1767)}$=12.3, p<0.001****; 95% CI 0.1 to 0.4****) (S1 Table S5)****). There was no statistical evidence of differences in completion rates across BMI categories, health literacy, snacking scores or habit strength (S1 Tables S4-S5).”***

4. Page 18 I think all the predictors of missingness should be in the model and I do not think they currently are.

   Response: Please see response to Comment 3 above – there was no statistical evidence of a difference for the remaining variables but these are now explicitly reported as such.

5. Page 24 While I agree with the main thrust of the first paragraph here I would question the use of the word still in the phrase ‘there was still evidence’.

   Response: the word ‘still’ has been removed.

6. Page 26 The sentence starting ‘there was no overall effect’ omits what it was effect on.

   Response: This has been clarified as below (p25)

THE UNIVERSITY OF SYDNEY

"There was no overall effect of allocation method ***on reduction in unhealthy snacking****, controlling for the effects of health literacy, action plan, age, language spoken at home, education, habit strength, and baseline snacking scores ($\chi^2(2)$ =3.27, p=0.19)."*

Points of more substance

1. Influential points: The issue of what to do with influential points is always a complex one unless there is a clear explanation of why they have occurred.
    a. Presumably it is the changes which are relevant her, not the baseline or follow–up values as that is what is being modelled?
    b. Are these values as plausible as the authors suggest? I am not that familiar with nutrition data but it looks as though they are comparable with the calorific intake I remember the track cyclist Chris Hoy reporting when he was training for the Olympics (and the current figures do not include non–snacks).
    c. Rather than excluding them what happens is the authors either treat them as missing and then impute them or if they winsorise the top https://en.wikipedia.org/wiki/Winsorizing .
    d. Excluding different numbers of people (eight on page 24 and twenty–four on page 27) is very data–driven and it would be better to identify a common group of suspicious observations across the analysis.

The problem is that the models are clearly heavily influenced by the subset of observations so those observations do need detailed attention.

Response: You are correct in pointing out that the key variable of interest is *change* in snacking scores, rather than absolute baseline or follow-up snacking scores. We have amended the manuscript to reflect this issue (22-23):
"Although no participants reported the maximum possible ***absolute change*** *in snacking score (630 serves) that* ***could be obtained*** *using the instrument, a small number of plausible but extreme values were observed (****differences of up to 430.5*** *serves of unhealthy snacks per week* ***across time points****)."*

We consulted diet and nutrition academics but they were not able to provide an estimated upper limit on the number of serves of snacks a person could plausibly consume. We recognise this as a limitation of the instrument. This is now described in the limitations section of the discussion (p28):
"We also acknowledge the inherent limitation of a once-off measure of snacking behaviour. This approach reduces participant burden and minimises the risk of missing data but the resulting estimates may be less accurate than those for electronic diaries."

After much consideration, we also felt that participants with extreme values should not be treated as missing, or winsorised, as it may be that this group behaves differently/ responds differently to the intervention. This is potentially important for targeting the intervention to those who will benefit most. It is possible that people with extreme snacking behaviours may need a different kind of intervention altogether.

In relation to the identification of two sets of influential outliers, this is due to analysis of one arm (N=398) and then three arms (N=1,177). We do acknowledge that there are alternative ways of addressing this issue. It was after much deliberation, and great difficulty in identifying suspicious or implausible snacking scores, that we decided to use a more data-driven approach in our sensitivity analysis.

2. Structure: I found the overall structure of the paper confusing. This is because the two aims are presented together. If I understand this correctly aim 1 is about trying to replicate whether the model holds in a new sample. Aim 2 is the comparison of what the trial was set up to do: compare the arms. The second seems more important to me as it extends the theory in a novel way but is in danger of being lost. At the very least I think we need clearer signposting as to what the two models are showing us. I would also consider putting aim 2 first and then introducing aim 1 as a test of fidelity. Another option would be to remove aim 1 to supplementary material but this might be a bit strong. Of course if I have not understood what aims 1 and 2 are doing then even more reason to try to clarify as other readers may be equally dim.

   Response: We acknowledge that the second aim is the one that extends the theory in a novel way. However, we feel that the current order of the aims will present a more logical flow for readers who have not read the preceding paper (PLOS ONE, https://journals.plos.org/plosone/article?id=10.1371/journal.pone.0209863). This is because the rationale for Aim 2 (evaluating different allocation methods) is contingent on a differential effect of health literacy on action plan effectiveness (Aim 1). To improve clarity we have:
   - **Changed the subheading for Aim 1 in the analysis and results sections to be more descriptive (changed from 'Confirmatory analysis' to 'Effects of health literacy and action plan'**)
   - **Added a footnote to Figure 1 (CONSORT flow diagram) to emphasise which arms are involved in Aims 1 and 2:** *"Note. **Aim 1 was assessed using Arm A2 only. Aim 2 was assessed using Arms A2, B and C.** Details and results relating to Arm A1 are not the focus of the research questions in this paper and are not be reported here."*

3. The unreported arm: In principle I think all the arms should be reported in one paper but that is more of an editorial decision. Given the confusion I have already confessed to about the two aims in the present version having a third one may be the straw which breaks the camel's back.

   Response: Analysis of the third aim would make the paper substantially longer than it currently is and answers a different research question. We feel this would detract from current findings and potentially cause confusion for the reader. For this reason our preference is to explore the unreported arm in a separate paper.

4. Summary: Despite the length of this review I do not have major concerns. Obviously the issue of what snacks those missing at follow–up were consuming is important but unknowable.

   Response: We thank the reviewer for their positive comments and useful feedback to improve this manuscript.

<u>Reviewer #2:</u>

The purpose of the study was to evaluate: 1) the impact of health literacy and action plan type on unhealthy snacking, for people who have type 2 diabetes or high BMI; and 2) the most effective method of action plan allocation. This is an interesting study but needs to be enhanced in terms of readability and other issues below.

1. The research problem, study design, analysis, and the results need to be stated in a consistent way.

THE UNIVERSITY OF SYDNEY

a. Hypothesis 1: The authors stated that there is a need to identify the effect of tailored action plan for people with low and high health literacy (p.5), but a recent study already showed the findings in the introduction (p.6). Then, what is the value of this study (hypothesis 1), in addition to the previous findings?

   Response: Our previous findings were novel and previously untested. This study strengthens the evidence that an individual's health literacy can impact behaviour change intervention effectiveness by demonstrating reproducibility. We have addressed this in the current study by testing the intervention in an independent, clinically relevant sample. We wanted to demonstrate evidence that the snacking intervention was relevant for people who have type 2 diabetes or overweight/obese BMI, as they stand to benefit more from such an intervention.

   We have revised the introduction to emphasise this (p6):
   These findings [from our previous study] contrast with the assumptions of the universal precautions approach and instead suggest that strategies to promote action may need to be tailored to a person's health literacy level. ***However, this is a novel area of research that warrants investigation in additional, varied samples, especially in light of the current reproducibility crisis across health and psychology*** **(Open Science Collaboration, 2015)***. The current paper aimed to build on the previous study in two ways: 1) to evaluate the impact of health literacy and type of action plan ('literacy-sensitive' or 'standard') on unhealthy snacking in a sample for whom reduced snacking would be of clinical benefit; and 2) to evaluate the effect of method of allocation to either a literacy-sensitive or standard action plan on overall effectiveness of the intervention***.**

   Open Science Collaboration. (2015). Estimating the reproducibility of psychological science. *Science, 349*(6251), aac4716. doi:10.1126/science.aac4716

b. Hypothesis 2: What is action plan allocation in terms of practical usefulness? Did the authors want to compare the effect of tailored action plan after HL screening with the patients' choice of preferred action plan? If so, subgroup comparison between the group received tailored action plan and the group preferred action plan would be sufficient. It seems that there is no need to compare these groups with random allocation group.

   Response: We acknowledge that they key comparison of interest was between the screened and choice arms. We agree that the random allocation arm is very unlikely to be selected for implementation. However, it serves as a control – for example, if either the screened or choice allocation method had proven detrimental for participants, these effects can be compared to the control (random allocation). If this had been the case it would highlight the need for further research into allocation methods that do not detract from the effects of the snacking intervention itself.

2. There might be no need to address about research questions not analyzed in this study (p.7, 3rd paragraph; p.8, line 5-7).

   Response: The additional research question (and additional arm) not analysed in this paper was included for completeness of reporting. To address this comment we have moved the final paragraph of the introduction (p8), which provides a description of

research question not analysed in this manuscript, into the discussion (p30), under the section on future directions:

"Lastly, it is unknown what impact assessment of participant preferences might have on action plan effectiveness. As outlined in our previously published protocol [33], this research question will be addressed by analysis of an additional arm not described in this paper (Arm A1; Fig 1). Analysis pertaining to this research question will be published elsewhere."

We have also updated the Methods to improve flow and clarity. This includes:

- **Changes to the Study Design section (p8):**
  *"This is a **two-stage** online randomized controlled trial that compared the effects of health literacy, action plan type, and method of allocation to an action plan **(random, screened and choice)** on self-reported unhealthy snacking over one month (Fig 1). **A third** research question involving Arm A1 in Fig 1 **(effect of assessment of preference), and preference and selection effects, as outlined in the protocol paper [33, 34]**, will be addressed elsewhere. This study was approved by the University of Sydney Human Research Ethics Committee [2018/793]. The study was prospectively registered with ANZCTR (www.anzctr.org.au) (ACTRN12618001409268)."*

- **Removal of the description of the additional arm from the Allocation Methods section (also under Methods, p11)**

3. Please describe the study design in detail. Please describe about reliability and validity of the measurement.

Response: A section titled 'Study design' is included on p8 (copied below, with tracked changes from other reviewer comments shown in bold). We also provide reference to our (open access) protocol paper describing the methods and measures in more detail, and refer to our (open access) publication reporting our initial findings which we sought to replicate (Aim 1). We do acknowledge that there are limitations of using once-off self-report measures. In line with revisions addressing Reviewer 1's 'Points of more substance' (1b), we have added this limitation in the discussion (p29) and abstract (p2). This limitation is also already highlighted in our published protocol.

> ***"Study Design***
> *"This is a **two-stage** online randomized controlled trial that compared the effects of health literacy, action plan type, and method of allocation to an action plan **(random, screened and choice)** on self-reported unhealthy snacking over one month (Fig 1). **A third** research question involving Arm A1 in Fig 1 **(effect of assessment of preference), and preference and selection effects, as outlined in the protocol paper [33, 34]**, will be addressed elsewhere. This study was approved by the University of Sydney Human Research Ethics Committee [2018/793]. The study was prospectively registered with ANZCTR (www.anzctr.org.au) (ACTRN12618001409268)."*
>
> **Discussion – section on limitations (p28):**
> *"We also acknowledge this study's limitations. We observed low-moderate retention (67%). This was unexpected given high retention in the previous study (85%) [21]. There was no difference in retention for people with low and high health literacy, but people with diabetes were less likely to complete follow-up, and this should be addressed in future research. Secondly, the action plan preferences of participants in*

the choice arm were informed by the names ("Smart Snacking 101 (basic)" and "Smart Snacking Pro (advanced)") and descriptions of our interventions. Whilst we intended these to accurately contrast the two action plans and present both in a positive light, these descriptions would have generated expectations and potentially influenced intervention appraisal and effectiveness. ***We also acknowledge the inherent limitation of a once-off measure of snacking behaviour. This approach reduces participant burden and minimises the risk of missing data, but the resulting estimates may be less accurate than those for electronic diaries."***

Abstract (p2): sentence added: "Key limitations include low-moderate retention and use of a single-occasion self-reported primary outcome."

4. Please report the results according to the research questions.

   Response: Our aims are stated on p6, and we believe that our results section has been structured in a way which adequately reflects these aims:

   "1) to evaluate the impact of health literacy and type of action plan ('literacy-sensitive' or 'standard') on unhealthy snacking in a sample for whom reduced snacking would be of clinical benefit; and 2) to evaluate the effect of method of allocation to either a literacy-sensitive or standard action plan on overall effectiveness of the intervention."

   To improve clarity and in line with our response to Reviewer #1's 'points of more substance' comment 2, we have also changed the subheading for Aim 1 in the analysis and results sections to be more descriptive (changed from 'Confirmatory analysis' to 'Effects of health literacy and action plan'). In the results section, following a general description of the sample, we present sections titled 'Aim 1: Effects of health literacy and action plan' and 'Aim 2: Effects of allocation method.'

<u>Reviewer #3: See attachment</u>

1. I would suggest adding more background literature on the "universal precautions" approach. This would provide the reader with more insight into what this approach is and how it has been used in health interventions.

   Response: We have added the following text in the introduction (p5):

   "The main approach to addressing low health literacy in the design of health information is to reduce the cognitive demand of ***all*** *health resources [6], which is most easily achieved by simplifying health information so that it is easier to understand* ***for everyone*** *[5-7].* ***Often this involves strategies such as using simple language and images, and chunking information into manageable pieces*** *[7]. However, little emphasis is placed on reducing the cognitive demand of behaviour change strategies that promote action such as planning, self-monitoring and problem solving [8, 9]. This represents an important research gap, particularly in the context of chronic conditions (including diabetes), as strategies that promote action play a key role in self-management behaviours such as healthy eating and physical activity [10-14].*

   In terms of implementation, this approach also recommends that all consumers can benefit from a health-literate intervention design [7]. This 'universal precautions' approach ***has shown to increase comprehension of health information and knowledge uptake for people with lower health literacy [5, 15-17]. It also*** *has practical advantages, because there*

is no need to assess a person's health literacy, nor to provide and distribute tailored interventions. However, it is important to consider that in applying a universal precautions approach to behavioural interventions, we implicitly assume that all consumers will benefit from strategies to promote action that are less cognitively demanding. This may not be the case; at least in some cases, cognitive effort has shown to increase engagement in these kinds of tasks [18, 19]. To date, few studies have evaluated whether applying health literacy principles to strategies promoting action ***might*** *have different effects for people with low and high health literacy [5, 15, 17, 20]."*

Methods:

a. *I would suggest explaining why participants with diabetes were recruited first followed by expanding the participant pool to people with who were considered overweight or obese.*

   Response: We recruited participants with diabetes first as people they made up a smaller proportion of the entire recruitment pool of participants compared to people with overweight/obese BMI. We wanted to recruit as many people with type 2 diabetes as possible within practical time constraints before opening the survey up to people who had overweight/obese BMI (who were much faster to recruit because they made up a larger proportion of the participant pool). As stated in the methods section 'Sample size' (p15), our a priori target sample size was 1,764 participants at baseline across the three arms involved in this paper. We have amended the 'Participants and recruitment' section of the methods (p8) to read:

   To maximise the proportion of the sample with type 2 diabetes*, people with diabetes were actively recruited first, after which the recruitment strategy was broadened to include those with overweight or obese BMI.*

b. In Figure 1 for the allocation method, I would encourage the authors to make sure the names of the allocation methods coincided with the allocation methods in the paper narrative. For example, in the paper, the allocation method is "Random (Arm A2)" but in the figure, the allocation method for is "Random (Arm A)". I would suggest adding the "2" so they would be the same. Please include the length of the intervention and how often did the participants have to engage in the intervention.

   Response: For clarity around only including participants in Arm A2 of the analysis, we have amended the text (see page 15; Aim 1: Effects of health literacy and action plan).

   We have also bolded 'Arm A2' in Fig 1 to make this more obvious p10) and revised the footnote in Fig 1 "Note. Aim 1 was assessed using Arm A2 only. Aim 2 was assessed using Arms A2, B and C. Details and results relating to Arm A1 are not the focus of the research questions in this paper and are not be reported here.**"**

c. Also, was race considered? If so, I would suggest including this information in your paper. When intervention materials were designed was health literacy considered? If so, please add why or why not. This could provide more insight on your findings.

THE UNIVERSITY OF SYDNEY

Response: Race was not considered in this analysis. In Australia, cultural diversity is often assessed by asking about a person's cultural background, whether the person was born overseas/ the number of years they've lived in Australia, or whether they speak English at home. We did include a variable 'speaks English at home' but the number of participants who did not speak English at home was very low (<2% of the sample). This is however a very interesting and important point, especially when it comes to changing eating behaviours. We are currently working with an Australian diabetes group to adapt the snacking intervention for people from an Indian cultural background. We have added an additional avenue of future research to investigate how the snacking tool could be optimised for people from different cultural backgrounds (p29):

"Given that low health literacy is more prevalent amongst people who speak English as a second language and who were born overseas [2, 5], this program of research could benefit from developing culturally-adapted versions of the snacking tool. This is particularly important given the influence of culture on diet [44, 45]."

With regards to the design on the intervention materials, we state on page 11:
"Literacy-sensitive action plan ("Smart Snacking 101 (basic)"): This intervention was designed for people with lower health literacy… Health literacy strategies such as simple language, white space and illustrative images were incorporated into the design [7]."

2. Discussion: Overall, very clear and connected to back findings. I would suggest discussing how the practical application of your results could aid in future intervention testing and clinical application.

   Response: Thank you for this positive feedback! In the discussion we elaborate on future directions of research that include future intervention testing (for example, testing health-literate designs for self-monitoring, and the additional example detailed above regarding culturally-adapted versions of the snacking tool. With regards to clinical application we have added the following text to the discussion (p28):

   In addition, the snacking plans are a low-cost and easy to use tool that could be feasibly incorporated into existing clinical practice.

3. Additional comments: The strengths of this article were the detailed approach to significance of health literacy and its impact on action planning with diabetic and overweight/obese individuals. The tables and figures were also well-detailed and connected to the research strategy. The weaknesses of this paper are that the authors did not address the ethnic/racial factors or barriers that individuals with low health literacy typically face when engaging in health related initiatives. Leaving the external generalizability of this study limiting. Overall, the authors did a great job of connecting each aim and discussing the importance of why health literacy should be taken into consideration. I would suggest minor revisions to enhance the quality of the paper.

   Response: Thank you for these positive comments about the strengths of the article. With regards to addressing ethnic/racial factors, we agree that this work is important. As

detailed above in response to comment 1c, we have identified adaptations as an important avenue for future work.

<u>Other minor additional changes (from authors)</u>

- We have made some minor changes to phrasing to improve expression and flow. These are shown in track changes in the manuscript.
- Please note that after correspondence with the Publishing Editor at PLOS Medicine, we are unable to show Fig 2 (reproduced from the protocol paper) as this will not adhere to the more restrictive CC BY-NC copyright required by BMJ Open. Fig 2 will be referenced accordingly as "Figure 2 in the protocol paper [35]".
