# Supplementary material for: Effects of health literacy, screening, and participant choice on action plans for reducing unhealthy snacking in Australia: A randomised controlled trial
Source: PLoS Med. 2020 Nov 3;17(11):e1003409. doi: 10.1371/journal.pmed.1003409 (PMC7608866; doi:10.1371/journal.pmed.1003409)
Supplement: Attachment — Submitted filename: response to reviewers (2nd round).pdf [file pmed.1003409.s008.pdf]

Dr Julie Ayre
Sydney School of Public Health, The University of Sydney
Tuesday, July 21st, 2020

Dear Dr Veitch,

RE: PLOS Medicine: Effects of health literacy, screening and participant choice on action plans for reducing unhealthy snacking: A randomised controlled trial" (PMEDICINE-D-20-00842R2)

Thank you again for the opportunity to revise our article submitted to PLOS Medicine. We have read the comments with interest, and revised our paper accordingly. We have addressed each editorial comment, with full details of the revisions given below in bold. These are also shown in tracked changes in the manuscript document. Line numbers below refer to numbers in the tracked version of the document. We have also checked to make sure images adhere to requirements and were uploaded to PACE. An open-access protocol has already been published (https://bmjopen.bmj.com/content/9/5/e028544) and is referred to in-text.

We trust that our adjustments make the paper suitable for publication in PLOS Medicine, and we look forward to your final decision.

Kind regards,

Julie Ayre,
On behalf of all authors

Comments from editors:

1. In general, the presentation needs to be significantly improved for publication in a medical journal and to more closely follow reporting for a clinical trial. For example, in the abstract no geographic, demographic, date information, intervention or outcomes are provided. These are all needed for trial publications. I have included more specifics below. Please do look at reporting on other trials for guidance. I am afraid the presentation issues are significant and should be resolved before we are able to proceed with your manuscript.

    a. When resubmitting please resubmit with line numbers.
    Line numbers have now been added to the manuscript. References to line numbers in this letter refer to those in the tracked changes version of the text.

    b. Title add a country setting, please.
    The title has been amended to include the country setting. The updated title is:
    "Effects of health literacy, screening and participant choice on action plans for reducing unhealthy snacking ***in Australia:*** *A randomised controlled trial"*

    c. In the abstract you mention the 'universal precautions approach' – firstly can you please state what this is, briefly (who signs up to it etc) and also please remove quote marks.
    The universal precautions approach has now been briefly described in the Background section of the abstract (p2 lines 19-26), with quotation marks removed as requested. The background section now reads

*“**Background:** Low health literacy is prevalent worldwide and is associated with poorer chronic disease outcomes.* ***A key strategy to address health literacy is a universal precautions approach, which recommends using health-literate design (e.g. simple language, images, and breaking information down into smaller, more manageable amounts) for all health interventions, not just those that target people with low health literacy. A universal precautions approach has practical advantages because there is no need to assess a person’s health literacy or tailor interventions. Further, this approach is widely endorsed internationally (e.g. by the World Health Organisation), and nationally (e.g. by the CDC and the Australian Commission for Safety and Quality of Healthcare).*** *However, action plans for behaviour change may be more effective when tailored by health literacy. This study evaluated: 1) the impact of health literacy and action plan type on unhealthy snacking, for people who have type 2 diabetes or high BMI; and 2) the most effective method of action plan allocation.”*

d. Abstract – please avoid a listy type of prose “Two-stage randomised controlled trial.” instead, we performed….
List-style prose has been removed as requested.

e. Abstract – and elsewhere, please be clear on what is statistically significant (with p values provided) and what’s not.
P-values have been added in the abstract (and in the main body text), where appropriate (see Methods and findings section abstract copied below for (Editor item 1f)). Please note that, for the point estimates that provide an example of the difference between the action plans (i.e. 10- and 3- point difference between the action plans, for people scoring 1 SD below and above the sample average health literacy, respectively), 95% confidence intervals have been added to give an indication of the uncertainty around these estimates. These have been included to demonstrate the interaction effect, rather than being planned pairwise comparisons and as such, P-values have not been included.

f. Abstract Methods and Findings: * Please ensure that all numbers presented in the abstract are present and identical to numbers presented in the main manuscript text. * Please include the study design, population and setting, number of participants, years during which the study took place, length of follow up, and main outcome measures (note you don’t list the outcomes as yet) * Please quantify the main results (with 95% CIs and p values). * Please include the important dependent variables that are adjusted for in the analyses. * Please include the actual amounts and/or absolute risk(s) of relevant outcomes (including NNT or NNH where appropriate), not just relative risks or correlation coefficients. (example for absolute risks: PMID: 28399126). * Please include a summary of adverse events if these were assessed in the study. Please ensure summary demographic information is added;* In the last sentence of the Abstract Methods and Findings section, please describe the main limitation(s) of the study's methodology.
The Abstract Method and Findings have been amended in line with these editorial requests. Numbers presented in the abstract have been checked for consistency with the main manuscript text. Study design, population and setting, number of participants, study years, follow-up period and main outcome measures have now been stated. The main results now include both 95% CIs and p-values where appropriate, with explicit statement of the variables controlled for in adjusted analyses. Adverse events were not assessed in this study; this is now explicitly

THE UNIVERSITY OF SYDNEY

mentioned in the abstract. Summary demographics have now been included, as have the limitations of the study methodology.

Although it is possible to derive NNT/NNH for continuous outcomes, the minimally-interesting difference required for these calculation is not known in the context of discretionary unhealthy snacks. As the purpose of this work was primarily to demonstrate that action plans are differentially effective across health literacy levels, we feel that calculation of these measures would be beyond the scope of this work and potentially misleading.

The Abstract Method and Findings (lines 31 to 60; with corrections in bold) now reads:

*"**Methods and Findings: We performed a** two-stage randomised controlled trial **in Australia between 14th February and 6th June 2019**. 1,769 participants **(mean age: 49.8 years (SD=11.7); 56.1% female (n=992); mean BMI: 32.9 (SD=8.7); 29.6% self-reported type 2 diabetes (n=523))** were randomised to one of three allocation methods (random, health literacy screening, participant selection); and one of two action plans to reduce unhealthy snacking (standard vs literacy-sensitive). Regression analysis evaluated the impact of health literacy (Newest Vital Sign: **NVS**), allocation method and action plan on reduction in self-reported serves of unhealthy snacks **(main outcome)** after **4-week follow-up**. **Analyses controlled for age, level of education, language spoken at home, diabetes status, habit strength, and baseline self-reported serves of unhealthy snacks. Average NVS score was 3.6 out of 6 (SD=2.0).** Participants reported consuming 25.0 serves of snacks **on average** per week at baseline (SD=28.0).*

*1,177 participants completed follow-up (**66.5%**). The literacy-sensitive action plan was more effective for participants with low-moderate health literacy, whereas the standard action plan was more effective for those with high health literacy **in adjusted analyses** (**action plan × health literacy (NVS) interaction**: b= -3.25, 95% CI: -6.55 to 0.05, **p=0.05**). **On average,** people scoring 1 SD below the mean for health literacy consumed 10.0 fewer serves per week using the literacy-sensitive action plan **(95% CI: 0.05 to 19.5)**, whereas those scoring 1 SD above the mean consumed 3.0 fewer serves using the standard action plan **(95% CI: -6.3 to 12.2)**. It is unclear which allocation method is more effective. Key limitations include low-moderate retention, use of a single-occasion self-reported primary outcome, **and reporting of a number of extreme, yet plausible, snacking scores, which rendered the interpretation of study results more challenging**. **Adverse events were not assessed in this study.***

***Conclusions:** Tailoring to health literacy may improve effectiveness of action plans, but costs of this strategy compared to universal precautions approach needs further investigation. This study highlights the importance of considering differential effects of health literacy on intervention effectiveness.*

Trial registration number ACTRN12618001409268"

2. Data - PLOS does not permit "data not shown or data is available on request (from the authors)." Please remove this claim, or do one of the following: a) If you are the owner of the data relevant to this claim, please provide the data in accordance with the PLOS data policy, and update your Data Availability Statement as needed. b) If the data not shown refer to a study from another group that has not been published, please cite personal

communication in your manuscript text (it should not be included in the reference section). Please provide the name of the individual, the affiliation, and date of communication. The individual must provide PLOS Medicine written permission to be named for this purpose. c) For any other circumstance, please contact me ASAP.

We have updated the Data Availability Statement (p34, line 328) to read "*De-identified data are available in S4 Appendix.*" An Excel spreadsheet with accompanying metadata has been uploaded as S4 Appendix.

3. "chunking information" – please amend to perhaps, breaking into smaller / manageable…

We have amended as follows (lines 110,11, p5):
*"Often this involves strategies such as using simple language and images, and **breaking** information **down** into **smaller, more** manageable **amounts**."*

4. "The current paper" – the current 'study'

This is now revised as above (line 161, p7)

5. Again, on presentation please do read published trials to see presentation – in the introduction we need more of the core information presented, interventions, outcomes and so on

In the introduction we have added more detail about the primary outcome, and a lengthier description of the literacy sensitive intervention (p6, lines 135 to 151):

*"Our recent randomized control trial (RCT) addressed both of these gaps in the literature [21]. We evaluated the impact of applying health literacy principles to an online action plan to reduce **self-reported** unhealthy snacking **over a 4-week period (where unhealthy snacking refers to non-alcoholic discretionary foods listed in the 'Australian Guide to Healthy Eating' [22]).** The 'literacy-sensitive' version of the action plan was compared to 'standard' action plan instructions that have been used previously in samples of the general population [23]. **The literacy-sensitive action plan incorporated health literacy strategies such as simple language, white space and illustrative images, and guided participants step-by-step to create an effective plan. This involved identifying a 'snack moment,' (a cue associated with snacking) and a solution (a behaviour to replace snacking when the cue occurred). The literacy-sensitive action plan asked participants to select plan characteristics from a predetermined list. By contrast,** the standard action plan asked participants to formulate their own free-text plans, **affording** greater flexibility and potential for personalisation. The results showed that the effectiveness of each plan depended on the participant's health literacy: people with lower health literacy reported consuming fewer unhealthy snacks at follow-up when they had used the literacy-sensitive action plan, whereas people with higher health literacy reported consuming fewer unhealthy snacks using the 'standard' action plan [21]."*

We have also added a TIDIER checklist as S1 Appendix (referenced page 8 line 203).

6. Page 9 – you say "Participants were excluded if they did not speak English, but they were not excluded on the basis of their snacking behaviour." And in Fig 1 you say exclusions were based on BMI. Im confused – can you please clarify / be consistent?

We have revised the statement to clarify (p9 lines 214-216): "*Participants were **not eligible** if*

they did not speak English. ***No participants*** *were excluded on the basis of their snacking behaviour* ***(e.g. participants with extreme, yet plausible, snacking scores were not excluded)****."*

The inclusion criteria for the study (p9 lines 207-209) were self-reported diagnosis of type 2 diabetes and/or self-reported height and weight corresponding to an overweight or obese BMI. To clarify Fig1, the phrase '*excluded based on BMI/diabetes status***' has been replaced with '*ineligible as did not have type 2 diabetes or high BMI*' (Fig1 p10).**

7. How is health literacy assessed / measured?

Health literacy was assessed using the validated Newest Vital Sign, as described in the abstract (line 36-37, p2) and methods (lines 287 to 294, p13)

Abstract: "*Regression analysis evaluated the impact of health literacy (Newest Vital Sign:* ***NVS****), allocation method and action plan on reduction in self-reported serves of unhealthy snacks* ***(main outcome)*** *after 4-week follow-up."*

Methods: *"Health literacy was measured using the Newest Vital Sign (NVS) [39]* ***(Cronbach's α =0.77),*** *a 6-item measure of functional health literacy, and a single-item literacy screener [40].* ***The NVS is a reliable instrument for assessing health literacy that has been validated through comparison to the Test of Functional Health Literacy in Adults (TOFHLA), a gold-standard measure of functional health literacy [40]."***

8. Please ensure all questionnaires are provided as Supp files.

This has now been added as a supplementary file (S2) and referred to on line 225 p9.

9. Snacking scores section – please be explicit – what is the primary outcome? I think you have used the header to tell us but we need it in sentence form.

This has now been clarified with the statement (lines 305-307, p 13-14):
"The primary outcome is *reduction in unhealthy snacking between baseline and follow-up (that is, a positive number indicates a reduction in the snacking score)."*

10. Secondary outcomes – again it is written in a listy way. Instead of writing the outcome as the header, with resulting multiple headers, please state in a sentence what the secondary outcomes are, how they're measured and what the follow up time is (and these should match your trial registration page). There should be one header "Secondary outcomes"

Thank you for this guidance we have now reformatted the secondary outcomes section (p14 lines 310-322) according to your advice:

"Difficulty using the planning tool was assessed using a 5-point scale (1=not at all hard, 5=extremely hard). This was assessed immediately following the intervention. This instrument was analysed as 3 categories: 'not at all hard'; 'a little hard; 'somewhat hard' to 'extremely hard.' Intention to reduce unhealthy snacking was also assessed immediately following the intervention, but not analysed at this timepoint in this paper. The remaining secondary outcomes were assessed at 4-week follow-up. *Perceived extents of healthy and unhealthy snacking in the previous week were each assessed using a single item 7-point Likert scale (from 1- not at all healthy/unhealthy; to 7 – very much healthy/unhealthy). Intention to reduce unhealthy snacking was assessed using three items [42, 43], habit strength was assessed using*

the 12-item self-report habit strength index [44], and action control ***(strategies to self-regulate unhealthy snacking)*** *was assessed using a 6-item measures [45]. Each of these latter three items was assessed using a 7-point Likert scale (strongly disagree to strongly agree).* ***Difficulty following the plan (likert scale ranging from 0 (very easy) to 10 (very hard)) and action plan preference were also assessed at 4-week follow-up but will be addressed elsewhere."***

11. All quantifiable data needs to have accompanying 95%Cis and p values and state is significant.

We have added 95% CIs and p values where appropriate (this includes in the Abstract (see changes outlined above), the point estimates for participants scoring 1 SD above/below mean health literacy score (Results section p20-21, lines 444-454), the confidence intervals on page 23 lines 480-483), to describe differences in mean health literacy score for participants in the choice arm (p25, lines 534-535). A P-value is now reported alongside each of the confidence intervals for effects of action plan, allocation method, health literacy (NVS), and diabetes status (p25, lines 540, 544, 550-551, p26 lines 559 and 556).

Furthermore, in the *Analysis* section of the Methods, we have added a statement to indicate "*For each of the following regressions, unadjusted and adjusted models are presented. P-values < 0.05 were considered statistically significant.*" (line 350 p 15).

Where results were descriptive in nature, we have ensured the language reflects this and does not imply a statistical difference (see for example, description of proportion of participants in each allocation method arm who used the literacy-sensitive plan (p24-25, lines 523-524). The original text made statements about higher/lower proportions:
"Approximately half of the participants in the randomized arm were assigned to use the literacy-sensitive action plan (N=302, 51.4%). ***For those in the choice arm*** *this proportion was* ***65.7%*** *(N=383),* ***whereas this was 44.6%*** *in the screened arm (N=267) (Table 1)."*

12. Results section – please go through this and compare to Table 1 – I find it very confusing. For example you say "1,769 participants at baseline, of whom 523…." And "Overall, 66.5% (N=1,177) of the participants completed follow-up" perhaps im confused, but this doesn't seem to match Figure 1. Also page 17 you say "more likely to report a diagnosis of type 2 diabetes" yet in Figure 1 those with diabetes are excluded and you also refer on pages 25 and 26 and elsewhere to those with diabetes.

We have double checked that numbers are correct, and acknowledge that it can be difficult to see the relationship between numbers in the figure and those reported in the text as there is no total (across all action plans and allocation methods) for participants included in either baseline or follow-up analyses. The totals for baseline are available from Table 1 (although there is not total column, in alignment with CONSORT guidelines). Totals for follow-up are available from S1 (now S3) Table S4. To clarify interpretation we have added the following text to the footnote of Fig 1 (p10 lines 232-233):

"Numbers indicated under 'type of action plan' sum to the total baseline sample who created an action plan and were included in the baseline sample (N=1,769). Numbers indicated under 'analysis' sum to number of participants who completed follow-up (N=1,177)."

In response to the comment about diabetes diagnoses, we hope that our changes resulting from Editor item 6 more clearly illustrate that the sample did include people who self-reported

a diagnosis of type 2 diabetes.

13. Please ensure that numbers in all tables match those given in Fig 1.

We have checked numbers reported in Table 1 and Fig 1. Again, to assist with interpretation of the table, we have added more detail to the footnote (p10 lines 232-233):

"*Numbers indicated under 'type of action plan' sum to the total baseline sample who created an action plan and were included in the baseline sample (N=1,769). Numbers indicated under 'analysis' sum to number of participants who completed follow-up (N=1,177).*"

14. CONSORT checklist – please use sections and paragraphs as page numbers change during revisions, etc.

This has now been updated. The CONSORT and TIDIER checklists are both now available from S1 Appendix and referenced under the Study Design section of the Methods (p8 line 203).

15. Did your study have a prospective protocol or analysis plan? Please state this (either way) early in the Methods section. a) If a prospective analysis plan (from your funding proposal, IRB or other ethics committee submission, study protocol, or other planning document written before analyzing the data) was used in designing the study, please include the relevant prospectively written document with your revised manuscript as a Supporting Information file to be published alongside your study, and cite it in the Methods section. A legend for this file should be included at the end of your manuscript. b) If no such document exists, please make sure that the Methods section transparently describes when analyses were planned, and when/why any data-driven changes to analyses took place. c) In either case, changes in the analysis-- including those made in response to peer review comments-- should be identified as such in the Methods section of the paper, with rationale.

We state on p 8 line 202-203 (Study Design section of the Methods) that the study was prospectively registered with ANZCTR (www.anzctr.org.au) (ACTRN12618001409268), and refer to the open access protocol paper on p 8 in the same paragraph (reference 38):
"This is a two-stage online randomized controlled trial that compared the effects of health literacy, action plan type, and method of allocation to an action plan (random, screened and choice) on self-reported unhealthy snacking over one month (Fig 1). A third research question involving Arm A1 in Fig 1 (effect of assessment of preference), and preference and selection effects, as outlined in the protocol paper [37, 38], will be addressed elsewhere. This study was approved by the University of Sydney Human Research Ethics Committee [2018/793]. The study was prospectively registered with ANZCTR (www.anzctr.org.au) (ACTRN12618001409268)."

All analyses were planned, including explicit description to include the covariates of health literacy (age, English as a second language and education) and baseline snacking. To improve transparency we have added a sentence to highlight that inclusion of habit strength as a covariate was not planned. This is outlined under Results for Aim 1 (page 20 lines 434-435):
"*Within the randomized arm, participants using the literacy-sensitive plan reported lower baseline habit strength compared to participants using the standard action plan (Table 2). Given that habit strength is an important theoretical moderator of the impact of this intervention [46, 47] and the small difference across action plan groups, this variable was included as a covariate in the regression models.* ***This covariate was not explicitly included in the analysis plan outlined in the registered trial information."***

16. Comments from the reviewers:
Reviewer #1: The authors have addressed my points.
As I originally mentioned the question of what to do about possible outliers is a complex one and we do still disagree about possible ways forward. Given the difficulties outlined in the rebuttal I would not want to push this but I would suggest that a sentence be added to the limitations stating that these people are a problem for interpretation.

We would like to acknowledge this issue in the limitations (and in the limitations listed in the abstract (see item 1 above)). This section of the discussion now reads (p28, lines 618 - 621):
"We also acknowledge this study's limitations. We observed low-moderate retention (67%). This was unexpected given high retention in the previous study (85%) [23]. There was no difference in retention for people with low and high health literacy, but people with diabetes were less likely to complete follow-up, and this should be addressed in future research. Secondly, the action plan preferences of participants in the choice arm were informed by the names ("Smart Snacking 101 (basic)" and "Smart Snacking Pro (advanced)") and descriptions of our interventions. Whilst we intended these to accurately contrast the two action plans and present both in a positive light, these descriptions would have generated expectations and potentially influenced intervention appraisal and effectiveness. We also acknowledge the inherent limitation of a once-off measure of snacking behaviour. This approach reduces participant burden and minimises the risk of missing data, but the resulting estimates may be less accurate than those for electronic diaries. ***Lastly, we acknowledge that the inclusion of participants with extreme values for self-reported unhealthy snacking renders interpretation more difficult. Given that these responses cannot be ruled out as impossible, we have provided a sensitivity analysis to maintain transparency of these findings."***
