# Supplementary material for: Effects of health literacy, screening, and participant choice on action plans for reducing unhealthy snacking in Australia: A randomised controlled trial
Source: PLoS Med. 2020 Nov 3;17(11):e1003409. doi: 10.1371/journal.pmed.1003409 (PMC7608866; doi:10.1371/journal.pmed.1003409)
Supplement: Attachment — Submitted filename: response to reviewers (3rd round).pdf [file pmed.1003409.s009.pdf]

THE UNIVERSITY OF SYDNEY

Dr Julie Ayre
Sydney School of Public Health, The University of Sydney
Tuesday, August 25th, 2020

Dear Dr Clare Stone,

RE: PLOS Medicine: Effects of health literacy, screening and participant choice on action plans for reducing unhealthy snacking: A randomised controlled trial" (PMEDICINE-D-20-00842R3)

Thank you again for the opportunity to revise and resubmit. We have addressed each editorial comment, with full details of the revisions given below in bold. These are also shown in tracked changes in the manuscript document. Line numbers below refer to numbers in the tracked version of the document.

With regards to the comment regarding claims of 'more effective' and 'greater reduction' we have tried to respond as best we can to take on this feedback. We are also happy to make further changes to more accurately reflect the true nature of the data if requested.

Kind regards,

Julie Ayre,
On behalf of all authors

Requests from the editors:

1. It seems issues persist around presentation. Repeatedly in the manuscript, there are claims of "more effective" and "greater reduction" and the like, but it may well be that there are in fact no statistically robust findings. Can you please respond to this and clarify in the manuscript on each occasion. Note, we will not continue with this manuscript if these issues persist as we have requested on more than one occasion.
   Throughout (Abstract, Author summary, Results, Discussion and Conclusion) we have further revised the manuscript to more accurately reflect the study findings as requested. Our revisions seek to place greater emphasis on estimates of the effects and their associated confidence intervals. We have also softened language around 'more effective' and 'greater reduction' where confidence intervals indicated that no effect may be present. We feel this approach better aligns with the 2016 ASA statement on p-values (https://doi.org/10.1080/00031305.2016.1154108) that suggests scientific conclusions should not be based only on whether a p-value passes a specific threshold. As in the previous version, we continue to report p values and now report exact p values, as requested. We hope this provides a more nuanced and precise description of the data.

   For example, with regards to the interaction effect for Aim 1 (action plan × health literacy (NVS) interaction) we have amended this in the following sections:
   Abstract (p2-3, lines 39-58):
   On average, people scoring 1 SD below the mean for health literacy consumed 10.0 fewer serves per week using the literacy-sensitive action plan (95% CI: 0.05 to 19.5), whereas those scoring 1 SD above the mean consumed 3.0 fewer serves using the standard action plan (95% CI: -6.3 to 12.2); ***however, we could not rule out the possibility of no action plan × health literacy (NVS) interaction (b= -3.25, 95% CI: -6.55 to 0.05, p=0.054)****.*

THE UNIVERSITY OF SYDNEY

<u>**Author summary (p4, lines 97-99):**</u>
The literacy-sensitive action plan ***may be*** *more effective for participants with low-moderate health literacy, whereas the standard action plan* ***may be*** *more effective for those with high health literacy*

<u>**Author summary (p4, lines 102-103):**</u>
There may be a benefit to tailoring actions plans to individual health literacy, particularly for those with lower health literacy.

<u>**Results (p20, lines 433-445):**</u>
Controlling for the effects of age, language spoken at home, education, habit strength, and baseline snacking scores, a *person who scored one standard deviation (2.0 points) below the mean NVS score (lower health literacy) would be predicted to consume 10.0 fewer serves of snacks per week using the literacy-sensitive action plan rather than the standard action plan (95% CI: 0.05 to 19.5). By contrast, a person who scored one standard deviation above the mean NVS score (higher health literacy) would be predicted to consume 3.0 fewer serves of snacks per week using the standard action plan, rather than the literacy-sensitive action plan (95% CI: -6.3 to 12.2) (Fig 2 Panel A).* ***However, we could not rule out the possibility of no true action plan × health literacy (NVS) interaction (b= -3.25, 95% CI: -6.55 to 0.05, p=0.054) (Table 3 and S3 Table S6).*** *Estimates from the intention-to-treat best- and worst-case scenarios were consistent with those from a complete case analysis (S3 Tables S7-8).* ***As shown in Fig 2, the relative benefit of the literacy-sensitive action plan compared to the standard action plan reversed as NVS scores moved beyond 5.***

<u>**Discussion (p27-28, lines 607-650):**</u>
This study found that the effectiveness of an action plan to reduce unhealthy snacking ***may*** *depend on the individual's health literacy level. In doing so, this study replicated a novel effect that has received little attention in the literature, in a sample of people with diabetes or overweight/obese BMI, for whom reducing unhealthy snacking is of clinical benefit****. On average, p****eople with lower health literacy reported* ***less*** *unhealthy snacking using an action plan that employed health literacy principles, whereas people with higher health literacy* ***appeared to*** *benefit more from using a 'standard' version of the action plan.* ***Although we could not rule out the possibility of no true interaction effect in the full sample, this effect was robust in our sensitivity analysis.*** *Results also suggested that the intervention overall was less effective for people with diabetes, and* ***that*** *screening for health literacy* ***could be*** *a more effective method of allocating an action plan than allowing the participant to select their preferred plan* ***(however the confidence intervals around the effect estimate for the latter observation do not preclude the possibility of not true difference). T****hese* ***last*** *two observations should be interpreted with caution as they were influenced by a small number of participants with extreme (yet plausible) values.*

This study raises questions about health literacy theory and intervention implementation. On the one hand, tailoring an action plan to a person's health literacy level ***may*** *improve plan effectiveness.* ***If this is the case, this*** *directly challenges the assumptions of a universal precautions approach to health literacy [7] (that is, that everyone benefits from simplified health materials), at least as it applies to a behaviour change action plan intervention. On the other hand, tailoring* ***appeared*** *to provide a substantial benefit for people with low to moderate health literacy* ***but*** *only a comparatively modest benefit for those with high health literacy. Given the aim of reducing health inequality, a universal precautions approach (i.e. only providing the literacy-sensitive action plan) may be more appropriate in this context than tailoring. A practical compromise may be to present the literacy-sensitive plan as the 'default' option, particularly as the benefits of different*

allocation methods (screening tool and allowing participants to choose) remains unclear.

Please also note, in the analysis section of the methods (p15 lines 339-340) we have now explicitly identified the process of considering influential observations as a 'sensitivity analysis' so that the revised text in the above section of the discussion is clear: "*We conducted a sensitivity analysis by* *sequentially* ***removing influential observations*** *individually from the model to check the robustness of the model*.**"**

<u>Conclusion (p30, lines 707-711):</u>
In conclusion, this study found that for people with diabetes or overweight/obese BMI, action plans to reduce unhealthy snacking ***may be*** *effective for people with lower health literacy when they employed health literacy principles. In contrast, 'standard' action plans that offered greater flexibility to personalise plans* ***may be*** *more effective for people with higher health literacy.*

The text below outlines further changes made in relation to Aim 2 (allocation method).
<u>Results (p25, lines 534-538)</u>
On average, participants in the screened allocation method reported a reduction of 1.79 serves of unhealthy snacks per week *compared to the choice allocation method****, though 95% confidence intervals suggest a true effect within the range of -0.16 to 3.73*** *(p=0.067) (Table 4 and S3 Table S12)*

<u>Discussion (p27, lines 615-621)</u>
Results also suggested that the intervention overall was less effective for people with diabetes, and ***that*** *screening for health literacy* ***could be*** *a more effective method of allocating an action plan than allowing the participant to select their preferred plan* ***(however the confidence intervals around the effect estimate for the latter observation do not preclude the possibility of no true difference).*** *These* ***last*** *two observations should be interpreted with caution as they were influenced by a small number of participants with extreme (yet plausible) values.*

<u>Discussion (p30, lines 699-706)</u>
Lastly, the findings from this study highlight the ***potential*** *limitations of conventional preference trial analyses. Overall, we found* ***limited*** *evidence that screening* ***may be*** *a more effective method of allocating an action plan than participant selection, but our exploratory findings suggest that a person's preference for an action plan may* ***also*** *play a role in action plan effectiveness. As such, further (a priori) analysis is warranted to estimate the independent contributions of a person receiving their preferred action plan and the effects of self-selection. An analysis that addresses this issue is outlined in the study protocol [38] and is in progress.*

<u>Conclusion (p30, lines 713-718)</u>
In addition, it remains unclear whether the intervention is more effective when plans are allocated through health literacy screening or allowing the individual to choose. ***Given the lack of a clear benefit of either allocation method, and a*** *relatively modest benefit of the standard action plan for people with higher health literacy, it may be more practical (and more cost-effective) to present the literacy-sensitive action plan as the 'default choice' rather than tailor to health literacy.*

2. Please trim the background subsection of the abstract

THE UNIVERSITY OF SYDNEY

In previous revisions, feedback requested that we describe the universal precautions approach and 'who signs up to it.' To balance this previous request with the current comment, we have retained the description of the universal precautions approach in the abstract but removed the statement that "*this approach is widely endorsed internationally (eg by the World Health Organisation), and nationally (e.g. by the CDC and the Australian Commission for Safety and Quality of Healthcare)*", along with some additional small editorial changes to keep language concise. The content of the deleted statement is still retained in the introduction.

The abstract background (p1, lines 18-26) now reads:
Background: Low health literacy is prevalent worldwide and is associated with poorer chronic disease outcomes. A key strategy to address health literacy is a universal precautions approach, which recommends using health-literate design (e.g. simple language ***and*** *images) for all health interventions, not just those that target people with low health literacy.* ***This*** *approach* ***is advantageous*** *because there is no need to assess a person's health literacy or tailor interventions. However, action plans for behaviour change may be more effective when tailored by health literacy. This study evaluated: the impact of health literacy and action plan type on unhealthy snacking, for people who have type 2 diabetes or high BMI* ***(Aim 1);*** *and the most effective method of action plan allocation* ***(Aim 2).***

3. Please rephrase "main outcome" as "primary outcome" in the abstract.
This has now been revised as requested (p2 line 34).

4. We suggest adapting the abstract to present the findings for "aim 1" and "aim 2" sequentially, reporting the number of participants and primary quantitative observations for each aim.
This is now revised as below in the methods and findings section of the abstract (p2-3 lines 39-64):
Aim 1: 398 participants **in the random allocation arm** completed follow-up **(67.7%).** On average, people scoring 1 SD below the mean for health literacy consumed 10.0 fewer serves per week using the literacy-sensitive action plan (95% CI: 0.05 to 19.5), whereas those scoring 1 SD above the mean consumed 3.0 fewer serves using the standard action plan (95% CI: -6.3 to 12.2)**; however, we could not rule out the possibility of no action plan × health literacy (NVS) interaction (b= -3.25, 95% CI: -6.55 to 0.05, p=0.054). Aim 2: 1,177 participants across the three allocation method arms completed follow-up (66.5%).** It is unclear which allocation method is more effective. Key limitations include low-moderate retention, use of a single-occasion self-reported primary outcome, and reporting of a number of extreme, yet plausible, snacking scores, which rendered the interpretation of study results more challenging. Adverse events were not assessed in this study.

5. Please rename figure 1 "Participant flowchart" or similar.
This has now been revised as suggested above (p10 line 229).

6. Around line 387, you describe "weak evidence" of a difference between groups in unhealthy snacking; these data appear to be those associated with a claim of "more effective" in the abstract, around line 45. Please ensure that the main findings are described in a consistent way - generally, rather than "weak evidence" we favour "non-significant reduction" or similar language.
These claims are now described in detail in a consistent way and described in detail in comment #1.

7. Please report exact p values or p<0.001 (noting the example at line 481).

All p-values are now provided as exact p-values (unless p<0.001), with all p-values <0.10 provided to 3 decimal places.

8. In the attached CONSORT and Tidier checklists, please refer to individual items by section and paragraph numbers rather than by line or page numbers.
   This is now revised as requested.
