# Supplementary material for: Effects of health literacy, screening, and participant choice on action plans for reducing unhealthy snacking in Australia: A randomised controlled trial
Source: PLoS Med. 2020 Nov 3;17(11):e1003409. doi: 10.1371/journal.pmed.1003409 (PMC7608866; doi:10.1371/journal.pmed.1003409)
Supplement: Attachment — Submitted filename: response to reviewers (4th round).docx [file pmed.1003409.s010.docx]

**Dear Dr Clare Stone and Dr Caitlin Moyer,**

**RE: PLOS Medicine: Effects of health literacy, screening and participant choice on action plans for reducing unhealthy snacking: A randomised controlled trial" (PMEDICINE-D-20-00842R4)**

**Thank you again for the opportunity to revise and resubmit. We have addressed each editorial comment, with full details of the revisions given below in bold. These are also shown in tracked changes in the manuscript document. We would also like to elect to publish the peer review history.**

**In addition we have incorporated changes requested in the “production issues” email (e.g. removing embedded figures, ensuring they are referenced in-text and have appropriate font size etc). All images and screenshots are now removed from S2 Appendix (survey). The data (S5) is reference now in the data availability statement**

1. Response to reviewer/editor comments: Thank you for your response that “Our revisions seek to place greater emphasis on estimates of the effects and their associated confidence intervals. We have also softened language around ‘more effective’ and ‘greater reduction’ where confidence intervals indicated that no effect may be present. We feel this approach better aligns with the 2016 ASA statement on p-values (<https://protect-au.mimecast.com/s/efmRCnx1jniVPNjEiN3L3q?domain=doi.org>) that suggests scientific conclusions should not be based only on whether a p-value passes a specific threshold. As in the previous version, we continue to report p values and now report exact p values, as requested. We hope this provides a more nuanced and precise description of the data.”

Please note that PLOS Medicine policy requires that findings be presented with both 95% CIs and p values. Further, in instances where confidence intervals (and p values) indicate that no effect is present, the language needs to convey that no significant effect was found. For example, in the author summary, “The literacy-sensitive action plan may be more effective for participants with low moderate health literacy, whereas the standard action plan may be more effective for those with high health literacy” is misleading as you did not observe a significant interaction effect. Please revise similar instances repeated throughout the manuscript.

**Thank you for this feedback. In addition to the items below we checked throughout the document that claims report p values and are accompanied by a statement about significance. In this process we identified one section that required additional reporting: we have added a table in S3 appendix (Table S16) to show the regression model for secondary outcomes for Research question 1. This is referenced in the section Aim 1>Secondary Outcomes>Perceived extent of unhealthy snacking (p22, line 485):**

*On average, participants in the randomized arm reported scores for perceived extent of unhealthy snacking that were 0.4 points higher on the scale (perceptions of greater extent of unhealthy snacking) at follow-up than at baseline (SD=3.22). Health literacy, action plan and diabetes status did not have significant effects on perceived extent of unhealthy snacking, controlling for the effects of age, language spoken at home, education, habit strength, and baseline perceived extent of unhealthy snacking* ***(S3 Table S16)****.*

2. Abstract: Methods and Findings: Line 33-36: Please also summarize the secondary outcome measures, and if space permits please present the findings (though we understand that word limits of abstract may preclude this).

**We have added the following text but as mentioned above are unable to add further detail due to word limits: *Secondary outcomes were perceived extent of unhealthy snacking, perceived difficulty using the plans (Aims 1-2), habit strength and action control (Aim 2).***

3. Abstract: Lines 39-44: For this sentence, please include p values in addition to the 95% CIs presented for the fewer servings per week consumed by those with 1SD lower than average health literacy using the literacy sensitive plan (please also specify that this is compared with the standard plan).

**We have revised as requested:**

*On average, people scoring 1 SD below the mean for health literacy consumed 10.0 fewer serves per week using the literacy-sensitive action plan* ***compared to the standard action plan*** *(95% CI: 0.05 to 19.5,* ***p=0.039****), whereas those scoring 1 SD above the mean consumed 3.0 fewer serves using the standard action plan* ***compared to the literacy-sensitive action plan*** *(95% CI: -6.3 to 12.2,* ***p=0.529****)*

4. Please include the p values for the fewer servings consumed by those 1SD above the mean health literacy score on the standard plan (and please also mention that this is in comparison to the literacy sensitive plan).

In addition to adding the p values, please do not use italics for emphasis, and we suggest revising to: “... however, we observed a non-significant no action plan × health literacy (NVS) interaction (b= -3.25, 95% CI: -6.55 to 0.05, p=0.054).” or similar to highlight that this interaction did not reach statistical significance.

**we have revised as requested above:** *however, we* ***observed a non-significant*** *action plan × health literacy (NVS) interaction*

5. Abstract: Line 45-46: “It is unclear which allocation method is more effective.” Please clarify what is meant by this sentence, e.g. by presenting your results.

**We have revised: *There was no effect of allocation method on reduction of unhealthy snacking, including no effect of health literacy screening compared to participant selection (b=1.79 95%CI: -0.16 to 3.73, p=0.067).***

6. Abstract: "Tailoring to health literacy may improve effectiveness of action plans, but costs of this strategy compared to universal precautions approach needs further investigation.”

This sentence does not appear to accurately summarize what can be concluded from your study. We suggest: “In our study we observed nominal improvements in effectiveness of action plans tailored to health literacy, however these improvements did not reach statistical significance, and the costs associated with such strategies compared with universal precautions needs further investigation.”

**We have revised as suggested above**

7. Author Summary: Why was this study done?: Please do not use italics for emphasis.

**Italics removed as requested**

8. Author Summary: What did the researchers do and find? Please revise the following points to accurately reflect your study (or similar):

--The literacy-sensitive action plan may be more effective for participants with low

moderate health literacy, whereas the standard action plan may be more effective for those with high health literacy. However, these effects did not reach statistical significance.

-- We did not find evidence suggesting that allocation had a significant effect on reported snacking behavior

**Revised as above**

9. Author summary: What do these findings mean?: Please revise the second bullet point to “However, this benefit did not reach statistical significance and should be weighed against the cost of tailoring compared to providing everyone with the same (literacy-sensitive) intervention (universal precautions approach).”

**Revised as above**

10. Introduction: Please consider shortening the Introduction section, particularly the information presented on pages 6-8. For example:

Lines 115-136: Please reduce the description of your previous study in the Introduction and summarize the findings more succinctly.

**We have revised as follows (p6, lines 137-148):**
*Our recent randomized control trial (RCT) addressed both of these gaps in the literature [23]. We evaluated the impact of applying health literacy principles to an online action plan to reduce self-reported unhealthy snacking over a 4-week period.* ***The ‘literacy-sensitive’ version of the*** *action plan incorporated health literacy strategies such as simple language, and images, and guided participants step-by-step to create an effective plan. This involved identifying a ‘snack moment,’ and a solution (a behaviour to replace snacking) from predetermined list****s****.* ***This was compared to a*** *‘standard’ action plan that asked participants to formulate their own free-text plans, affording greater potential for personalisation. The results showed that the effectiveness of each plan depended on the participant’s health literacy: people with lower health literacy reported consuming fewer unhealthy snacks at follow-up when they had used the literacy-sensitive action plan, whereas people with higher health literacy reported consuming fewer unhealthy snacks using the ‘standard’ action plan [23].*

Lines 141-147: The information in this paragraph could be made more succinct, or presented elsewhere.

**We have revised as follows (p6 lines 174-178):** *For people with type 2 diabetes and/or and overweight or obese body mass index (BMI), reducing unhealthy snacking is of clinical benefit and* ***may assist with weight management [25-27].*** *Weight management has shown to improve insulin sensitivity, fasting blood glucose, blood pressure and lipid levels, and help reduce the need for diabetes medications [28-30].*

11. Introduction: Line 160-167: When presenting your question or hypothesis, please use paragraph format rather than a numbered list.

**Revised to use paragraph format as requested.**

12. Methods: Line 176: Prospective plan- Please include the study protocol document and analysis plan, with any amendments, as Supporting Information to be published with the manuscript if accepted.

**We have added a protocol to S1 (CONSORT and TIDIER checklists), including mention of any amendments. This is now referenced in the section on ‘Study design’:** *This is a two-stage online randomized controlled trial that compared the effects of health literacy, action plan type, and method of allocation to an action plan (random, screened and choice) on self-reported unhealthy snacking over one month (Fig 1). A third research question involving Arm A1 in Fig 1 (effect of assessment of preference), and preference and selection effects, as outlined in the protocol paper [35, 36] (****see also*** ***S1****), will be addressed elsewhere. This study was approved by the University of Sydney Human Research Ethics Committee [2018/793]. The study was prospectively registered with ANZCTR (www.anzctr.org.au) (ACTRN12618001409268).* ***The protocol,*** *CONSORT and TIDIER checklists are* ***also*** *shown in S1.*

13. Results: Lines 383- 390: Please provide the p values to accompany the 95% CIs for the following results: “Controlling for the effects of age, language spoken at home, education, habit strength, and baseline snacking scores, a person who scored one standard deviation (2.0 points) below the mean NVS score (lower health literacy) would be predicted to consume 10.0 fewer serves of snacks per week using the literacy-sensitive action plan rather than the standard action plan (95% CI: 0.05 to 19.5). By contrast, a person who scored one standard deviation above the mean NVS score (higher health literacy) would be predicted to consume 3.0 fewer serves of snacks per week using the standard action plan, rather than the literacy-sensitive action plan (95% CI: -6.3 to 12.2) (Fig 2 Panel A).

**We have added the p values to accompany the 95% confidence intervals in this paragraph:**

*Controlling for the effects of age, language spoken at home, education, habit strength, and baseline snacking scores, a person who scored one standard deviation (2.0 points) below the mean NVS score (lower health literacy) would be predicted to consume 10.0 fewer serves of snacks per week using the literacy-sensitive action plan rather than the standard action plan (95% CI: 0.05 to 19.5,* ***p=0.039****). By contrast, a person who scored one standard deviation above the mean NVS score (higher health literacy) would be predicted to consume 3.0 fewer serves of snacks per week using the standard action plan, rather than the literacy-sensitive action plan (95% CI: -6.3 to 12.2****, p=0.529****) (Fig 2 Panel A).*

*14. Results: Lines 390-392: Please rephrase this sentence to read: “However,* *we did not observe a statistically significant action plan × health literacy (NVS) interaction (b= -3.25, 95% CI: -6.55 to 0.05,p=0.054) (Table 3 and S3 Table S6).*

**Revised as above**

15. Results: Lines 393-395: Please revise this sentence as follows to note that confidence intervals are overlapping/there is no statistically significant reversal. “ As shown in Fig 2, the relative benefit of the literacy-sensitive action plan compared to the standard action plan is apparently reversed as NVS scores moved beyond 5; however, the confidence intervals are overlapping” or similar.

**Revised to :** As shown in Fig 2, the relative benefit of the literacy-sensitive action plan compared to the standard action plan reversed as NVS scores moved beyond 5, **though note the confidence intervals are overlapping.**

16. Results: Please revise to include the result: “However, this effect was not robust to removal of outlier and influential observations (b=-0.82, 95%CI= (-5.07-3.43); p= 0.706) (S3 Table S9).

**Revised as above. Statistical reporting was amended to be a consistent format with other coefficients presented in the paper i.e. *(b=-0.82, 95%CI: -5.07 to -3.43, p= 0.706)***

17.Results: Line 437: Please indicate that “M” stands for “mean” at the first use in the text

**The first instance is in the second paragraph of results so we have explained this abbreviation there (p17, line 394):**

*There were 1,769 participants at baseline, of whom 523 (29.6%) reported a diagnosis of type 2 diabetes (Table 1). Across the three arms, participants were an average 49.8 years of age (SD=11.7), 56.1% were female (n=992), and average BMI was in the obese range (BMI >30) (****Mean (M)****=32.9, SD=8.7).*

18. Results: Lines 469-473: Please revise to “On average, participants in the screened allocation method reported a reduction of 1.79 serves of unhealthy snacks per week compared to the choice allocation method, though this did not reach statistical significance and 95% confidence intervals suggest a true effect within the range of -0.16 to 3.73 (p=0.067) (Table 4 and S3 Table S12).”

**Revised as above**

19.Results: Lines 488-490: Please revise to: “The resulting regression model coefficients are consistent with the initial analysis (‘All observations’, Table 4), but indicate a much smaller mean difference between participants in the screened and choice arms and did not reach statistical significance (Table 4 and S3 Table S15).” Please also provide results, 95% CIs and p values for the findings for the screened vs. choice comparison, as well as the finding for the main health literacty effect, for the analysis with outliers removed as these were highlighted for the “all data” analysis presented above.

**Revised as above:** *The resulting regression model coefficients are consistent with the initial analysis (‘All observations’, Table 4) but indicate a much smaller mean difference between participants in the screened and choice arms* ***and did not reach statistical significance (b=0.45 95%CI: -0.71 to 1.59, p=0.432)*** *(Table 4 and S3 Table S15). The main effects of allocation method* ***(χ²(2) =1.21, p=0.54)****, health literacy* ***(b=0.16, 95%CI: -0.35 to 0.67, p= 0.553)*** *and diabetes status (b= -0.32, 95% CI: -2.54 to 1.90, p=0.763) were not robust to removal of outlier and influential observations.*

20. Results: Lines 494-495: Please present the results in the text (with 95% CIs and p values) or provide a note to which table contains these findings. “There were no main effects of allocation method or diabetes status on perceived extent of unhealthy snacking, habit strength or action control.”

**In the manuscript we now reference new appendix tables (S3 Tables S17-S19) that provide these results.**

21.Discussion: Please revise throughout to reflect the study’s findings. Specifically, your results and statistical analyses do not support these conclusions. “This study found that the effectiveness of an action plan to reduce unhealthy snacking may apparently depend on the individual’s health literacy level; however, these findings did not reach statistical significance. The direction of this relationship is consistent with a novel effect that has received little attention in the literature, in a sample of people with diabetes or overweight/obese BMI, for whom reducing unhealthy snacking is of clinical benefit. On average, people with lower health literacy reported less unhealthy snacking using an action plan that employed health literacy principles, whereas people with higher health literacy appeared to benefit more from using a ‘standard’ version of the action plan. Although the interaction effect did not reach statistical significance in the full sample, this effect

was significant in our sensitivity analysis. Results also suggested that the intervention overall was less effective for people with diabetes, and that screening for health literacy could be a more effective method of allocating an action plan than allowing the participant to select their preferred plan; however the effect estimate for the latter observation did not reach statistical significance. These last two observations should be interpreted with caution as they were influenced by a small number of participants with extreme (yet plausible) values.

**Revised as above with a very slight change – as per comment 25 below, we have rephrased the sample description (people with diabetes or overweight/obese BMI) to ‘people with overweight/obese BMI or diabetes’ as the majority of the sample (>95%) had an overweight/obese BMI, in line with the eligibility criteria (p26, lines 551-566):**

*This study found that****, in a sample of people with overweight/obese BMI or diabetes****, the effectiveness of an action plan to reduce unhealthy snacking may* ***apparently*** *depend on the individual’s health literacy level;* ***however, these findings did not reach statistical significance****.* ***The direction of this relationship is consistent with*** *a novel effect that has received little attention in the literature,* ***and for a group*** *for whom reducing unhealthy snacking is of clinical benefit. On average, people with lower health literacy reported less unhealthy snacking using an action plan that employed health literacy principles, whereas people with higher health literacy appeared to benefit more from using a ‘standard’ version of the action plan****. Although the interaction effect did not reach statistical significance in the full sample, this effect was significant in our sensitivity analysis****. Results also suggested that the intervention overall was less effective for people with diabetes, and that screening for health literacy could be a more effective method of allocating an action plan than allowing the participant to select their preferred plan; however the effect estimate for the latter observation* ***did not reach statistical significance****. These last two observations should be interpreted with caution as they were influenced by a small number of participants with extreme (yet plausible) values.*

**Please also note, we have revised the paragraph in the discussion about strengths (p26) to be consistent with the above text (lines 593 to 600):**

*This study has several strengths.* ***The direction of findings was consistent with*** *our original findings in a more applied sample for whom reduced snacking would be of clinical benefit [23]. This study also used a validated instrument that has high relevance to the Australian context as it maps directly to national guidelines for healthy eating [39]. Furthermore, overconsumption of the unhealthy snacks targeted in this study have been identified as a key challenge to diet quality in Australia [39]. In addition, the snacking plans are a low-cost and easy to use tool that could be feasibly incorporated into existing clinical practice.*

22.Discussion: Lines 518-520: Please revise this sentence to more accurately reflect the findings, namely that the interaction between choice/screening and health literacy approached but did not achieve statistical significance. “On the other hand, tailoring appeared to provide a substantial benefit for people with low to moderate health literacy but only a comparatively modest benefit for those with high health literacy; however, the interaction between health literacy and using a screening approach approached but did not achieve statistical significance and were not robust to the removal of outliers.” or similar.

**Thank you for this feedback, we have used phrasing that mirrors your suggested text in item #21 (suggested wording there was: although the interaction effect did not reach statistical significance in the full sample, this effect was significant in our sensitivity analysis). We have also more fully expanded on the final sentence of that paragraph to make the results of the screening allocation method clearer. The text is revised as follows (p26-27 lines 574 – 592):**

*This study raises questions about health literacy theory and intervention implementation. On the one hand, tailoring an action plan to a person’s health literacy level may improve plan effectiveness. If this is the case, this directly challenges the assumptions of a universal precautions approach to health literacy [7] (that is, that everyone benefits from simplified health materials), at least as it applies to a behaviour change action plan intervention. On the other hand, tailoring appeared to provide a substantial benefit for people with low to moderate health literacy but only a comparatively modest benefit for those with high health literacy.* ***However, these findings were not definitive: the interaction effect, which would support the argument for tailoring, approached but did not achieve statistical significance in the full sample but was significant in our sensitivity analysis. A universal precautions approach (i.e. only providing the literacy-sensitive action plan) may be more appropriate in this context than tailoring****, given the aim of reducing health inequality.* ***Further, in terms of implementation, the effect of screening for health literacy approached but did not achieve statistical significance in the full sample and was not significant in our sensitivity analysis.*** *A practical compromise may be to present the literacy-sensitive plan as the ‘default’ option.*

23.Discussion: Lines 575-579: As this presumably refers to the arm of your study to be analyzed and published separately, please remove this from the discussion: “... but our exploratory findings suggest that a person’s preference for an action plan may also play a role in action plan effectiveness.” and please revise the following to: “Further (a priori) analysis is warranted to estimate the independent contributions of a person receiving their preferred action plan and the effects of self-selection.”

**Revised as follows:** *Lastly, the findings from this study highlight the potential limitations of conventional preference trial analyses. Further (a priori) analysis is warranted to estimate the independent contributions of a person receiving their preferred action plan and the effects of self-selection.*

24.Discussion: Limitations section: Please comment on the limitation that you did not achieve the goal of 500 participants per arm and the impact this may have on detecting statistically significant effects.

**Revised (p27 lines 601-607):** *We also acknowledge this study’s limitations. We observed low-moderate retention (67%). This was unexpected given high retention in the previous study (85%) [23]. There was no difference in retention for people with low and high health literacy, but people with diabetes were less likely to complete follow-up, and this should be addressed in future research.* ***Further, low-moderate retention reduced the number of participants in each arm available for per protocol analysis and may have reduced power to detect statistically significant effects.***

25. Discussion: Lines 581-585: Please revise to more accurately reflect the study findings. In particular, results were not presented suggesting effects in those with overweight/obese BMI: “In conclusion, this study found that the apparent directions of effects suggesting that for people with diabetes, action plans to reduce unhealthy snacking may be more effective for people with lower health literacy when they employed health literacy principles. In contrast, ‘standard’ action plans that offered greater flexibility to personalise plans may be more effective for people with higher health literacy. However, these effects should be interpreted with caution as they did not reach statistical significance.”

**The eligibility criteria for the study were self-reported diagnosis of type 2 diabetes and/or self-reported height and weight corresponding to an overweight or obese BMI (i.e., BMI ≥ 25 kg/m2), or a self-reported diagnosis of type 2 diabetes. As such, it was possible that some people diagnosed with type 2 diabetes did not have an overweight or obese BMI. However, diabetes is associated with higher BMI, and in our sample more than 95% of participants reported heights and weights corresponding to overweight or obese BMI (this is shown in Table 1). For this reason we felt it would be accurate to suggest effects in people with overweight/obese BMI.**

**To emphasise that the sample is mostly made up of people with overweight/obese BMI, throughout the manuscript we have revised ‘people with diabetes or overweight/obese BMI’ to ‘people with overweight/obese BMI or diabetes.’ This also reflects the fact that the majority of the sample was people who had an overweight/obese BMI but who did not have type 2 diabetes.**

**The revised section is as follows (all other revisions requested above are incorporated) p29 lines 657-662):**

*In conclusion, this study found* ***an apparent direction of effects suggesting that*** *for people with overweight/obese BMI* ***or diabetes****, action plans to reduce unhealthy snacking may be more effective for people with lower health literacy when they employed health literacy principles. In contrast, ‘standard’ action plans that offered greater flexibility to personalise plans may be more effective for people with higher health literacy.* ***However, these effects should be interpreted with caution as they did not reach statistical significance.***

26.Discussion: Lines 587-592: Please also revise to clarify these sentences: “In addition, we did not observe significant differences in effects on snacking when interventions are allocated through health literacy screening or allowing the individual to choose. Given the lack of a clear benefit of either allocation method, and the absence of a statistically robust benefit of the standard action plan for people with higher health literacy, it may be more practical (and more cost-effective) to present the literacy-sensitive action plan as the ‘default choice’

rather than tailor to health literacy.” Or similar.

**Revised as above.**

27. Data Availability Statement: This section can be removed from the main text of the manuscript. It will be extracted from the “Data Availability” section of the manuscript submission form, so please be sure all information there is entered accurately and completely.

**This is now removed**

28.Checklists: Thank you for including the CONSORT and TIDieR checklists; please remove references to page numbers as these are subject to change. Please instead only use section and paragraphs to refer to locations in text.

**This has been revised. Where there is only a single paragraph for a section, only the section is given.**

29. Figure 2: In the legend, please describe the differences between the two panels. For Panel A: On the Y axis, the negative signs are missing between 10 and 25 for the values between “no change” and “more snacking”.

**Differences between the two panels are identified through the panel titles (A: All observations and B: Outlier and influential observations removed). This mirrors the corresponding table subheadings. The issues with the negative signs have been corrected.**

30. Table 3: There is an extra common in the adjusted results 95% CIs presented for action plan in the top panel (3.53 (-3.12, 10.18,).

**Corrected.**
